# A «Repertoire for Repertoire» Hypothesis: Repertoires of Type Three Effectors are Candidate Determinants of Host Specificity in *Xanthomonas*

**DOI:** 10.1371/journal.pone.0006632

**Published:** 2009-08-14

**Authors:** Ahmed Hajri, Chrystelle Brin, Gilles Hunault, Frédéric Lardeux, Christophe Lemaire, Charles Manceau, Tristan Boureau, Stéphane Poussier

**Affiliations:** 1 Département Santé des Plantes et Environnement, Institut National de la Recherche Agronomique (INRA), Beaucouzé, France; 2 Département d'Informatique, Université d'Angers, Angers, France; 3 Département de Biologie, Université d'Angers, Angers, Beaucouzé, France; 4 Département de Sciences Biologiques, Agrocampus Ouest centre d'Angers, Institut National d'Horticulture et de Paysage (INHP), Beaucouzé, France; University of Wisconsin-Milwaukee, United States of America

## Abstract

**Background:**

The genetic basis of host specificity for animal and plant pathogenic bacteria remains poorly understood. For plant pathogenic bacteria, host range is restricted to one or a few host plant species reflecting a tight adaptation to specific hosts.

**Methodology/Principal Findings:**

Two hypotheses can be formulated to explain host specificity: either it can be explained by the phylogenetic position of the strains, or by the association of virulence genes enabling a pathological convergence of phylogenically distant strains. In this latter hypothesis, host specificity would result from the interaction between repertoires of bacterial virulence genes and repertoires of genes involved in host defences. To challenge these two hypotheses, we selected 132 *Xanthomonas axonopodis* strains representative of 18 different pathovars which display different host range. First, the phylogenetic position of each strain was determined by sequencing the housekeeping gene *rpoD*. This study showed that many pathovars of *Xanthomonas axonopodis* are polyphyletic. Second, we investigated the distribution of 35 type III effector genes (T3Es) in these strains by both PCR and hybridization methods. Indeed, for pathogenic bacteria T3Es were shown to trigger and to subvert host defences. Our study revealed that T3E repertoires comprise core and variable gene suites that likely have distinct roles in pathogenicity and different evolutionary histories. Our results showed a correspondence between composition of T3E repertoires and pathovars of *Xanthomonas axonopodis*. For polyphyletic pathovars, this suggests that T3E genes might explain a pathological convergence of phylogenetically distant strains. We also identified several DNA rearrangements within T3E genes, some of which correlate with host specificity of strains.

**Conclusions/Significance:**

These data provide insight into the potential role played by T3E genes for pathogenic bacteria and support a “repertoire for repertoire” hypothesis that may explain host specificity. Our work provides resources for functional and evolutionary studies aiming at understanding host specificity of pathogenic bacteria, functional redundancy between T3Es and the driving forces shaping T3E repertoires.

## Introduction

Deciphering the mechanisms used by bacterial pathogens to evolve and adapt to new hosts is a major issue for both medical and plant sciences. Despite the tremendous achievements of 30 years of intensive research and the mass of information provided by the sequenced genomes in understanding the interactions between bacterial pathogens and their hosts, the molecular factors underlying host specificity of pathogenic bacteria still remain to be identified. Such fundamental knowledge tackles both the co-evolution between the host and the pathogen, as well as the factors underlying the emergence of new pathogens. For example, recent studies investigated the similarities between human and avian pathogenic strains of *Escherichia coli*, to gain insight into potential zoonotic risks of avian pathogenic strains [1]–[5]. Indeed, strains of *E. coli* belonging to the same phylogenetic groups may display pathogenicity either on poultry or on humans, and the question whether avian strains may serve as potential reservoir of antibiotic resistance or virulence genes is of crucial importance [2].

For plant pathogenic bacteria, host specificity of strains is usually very high and well characterized. In many species of plant pathogenic bacteria numerous pathovars are defined. A pathovar is a subspecific division that groups all bacterial strains that cause the same symptoms on the same plant host range [6]. Within a pathovar, a second level of specificity is defined: races were defined based on the observation that some strains, although fully pathogenic on most host cultivars, may reveal avirulent on a certain cultivar. Such specificity between bacterial races and cultivars follows Flor's « gene for gene » theory [7], and has been largely exploited in crop breeding. In the last 20 years, examples accumulated of bacterial plant pathogens bypassing monogenic resistances introduced in crops. By contrast, host jumps only seldom occur, suggesting that host specificity barriers are more difficult to bypass. Therefore, understanding the molecular mechanisms underlying host specificity may lead to engineering more durable resistances for crop protection.

In the last ten years, genomes of many animal and plant pathogenic bacteria were completely sequenced. Comparative genomics studies demonstrated that repertoires of virulence associated genes can be highly variable among the sequenced strains, thus suggesting a role in host specificity [8]–[14]. However, despite the increasing number of microbial genomes available, genome comparison of several model strains may not fully represent the extreme variability of host specializations that can be found within one bacterial genus. For example, the *Xanthomonas* genus is constituted of 27 species causing diseases on more than 400 different host plants, among which many economically important crops [15]. At present, genomes of only 12 model strains belonging to diverse species and pathovars of *Xanthomonas* are sequenced or on the way to be sequenced (http://www.genomesonline.org/). As long as at least one strain representative of each pathovar is not sequenced, comparative genomics, although highly informative, is not fully suited for the identification of the molecular determinants of host specificity.

A pioneer study by Sarkar and colleagues [16] determined the distribution of a large scope of virulence associated genes in a collection of 91 strains of the plant pathogenic bacterium *Pseudomonas syringae*, isolated from diverse host plants. They postulated that looking at the distribution of these virulence-associated genes may provide clues on their possible role in host specificity: genes that are highly conserved among all strains, irrespective to the host of isolation, probably do not play a major role in host specificity. On the contrary, virulence-associated genes heterogeneously distributed among strains are good candidates to explain host specificity.

Among pathogenicity determinants shown to display heterogeneous distribution between strains are Type III Effectors (T3Es) [12], [16]. T3Es are bacterial proteins that are directly injected inside the cytoplasm of the host cell by a bacterial molecular apparatus called Type III Secretion System (T3SS). This system is conserved among most of the gram negative plant and animal pathogenic bacteria. Using the T3SS, each bacterial strain can inject up to 30 T3Es in the eukaryotic host cell simultaneously [17], [18]. In *Xanthomonas*, a mutation in the T3SS impairs the ability to inject T3Es in the host plant, and as a consequence abolishes pathogenicity and multiplication *in planta* [19]. More precisely, many studies demonstrated that T3Es alter the physiology of the host cell in a way that is beneficial for the pathogen [11], [20].

In 2006, Jones and Dangl [21] proposed a mechanistic model for the interaction between gram negative bacteria and plants in which the combined action of T3Es leads to suppression of host defence reactions that are induced after recognition of Pathogen Associated Molecular Patterns (PAMPs), thus resulting in an effector-triggered susceptibility. In some cases, individual T3E (or its action inside the cell) may specifically be recognized by the plant. This recognition induces a hypersensitive response (HR), resulting in an effector induced resistance. Such specific recognition of a T3E in the host cell by the product of a plant resistance gene constitutes the molecular basis of the “gene for gene” theory ruling race/cultivar specificity. Thus, T3Es may enlarge the host range of a given bacterium by suppressing host defences, or narrow the host range when one of them is specifically recognized by the plant.

Experimental data suggest that T3Es may also be involved in host specificity. Indeed, several studies used suppression subtractive hybridization (SSH) approaches to perform genomic comparison of non-sequenced strains, that are very closely related phylogenetically but differing in the hosts they attack. Among the genes isolated in these SSHs were single T3Es, suggesting that they can play a role in host specificity in plant pathogenic bacteria [22]–[24] as well as in animal pathogenic bacteria [2].

Regarding the different T3Es injected in the host cell, they probably act collectively. Supporting this idea, mutations achieved in one single T3E rarely affect the virulence phenotype of strains. This suggests that among all the T3Es injected in the host cell, some have redundant functions virulence [25], [26]. T3Es seem to act synergistically or antagonistically on different pathways of the host cell, to create a physiological status of the host that would be optimal for the development of the pathogen. Examples illustrating such idea can be found in plant and animal pathogenic bacteria. Among the conserved T3Es of *Pseudomonas syringae* pv. *tomato* DC3000, HopPtoM induces an increase of the number and the size of the lesions, whereas HopPtoN induces a decrease of the number and the size of the lesions [27], [28]. In *E. amylovora*, similar antagonism can be found also between HrpN and HrpW. Indeed, HrpN induces ion fluxes that induce cell death in *Arabidopsis*, whereas HrpW induce ion fluxes that prevent cell death induced by HrpN. When both purified proteins HrpW and HrpN are added in the culture medium, the quantity of cell death depends on the relative concentration of the proteins [29]. In *Salmonella typhimurium*, the T3E AvrA stabilizes cell permeability and tight junctions in epithelial cells, whereas the T3Es SopB, SopE and SopE2 were shown to destabilize tight junctions [30].

Moreover, it was shown that in maize, resistance genes involved in race/cultivar specificity may also play a role in the recognition of the non-host bacterium *Xanthomonas oryzae* [31]. This shows that the same type of genes may be involved at both levels, race/cultivar resistance and non-host resistance. Host specificity is most probably not governed by a unique gene. If so, emergence of new plant diseases would be as frequent as cases of bacterial pathogens escaping monogenic resistances. May we generalize the model described by Jones and Dangl [21], the outcome of the interaction would depend greatly on the confrontation of repertoires of T3Es and “guard genes” of the plant. Race/cultivar specificity would then be explained in a “Gene for Gene” manner [7] whereas host specificity would be explained in a “Repertoire for Repertoire” manner.

In the present study, we have chosen, as a model for investigating the molecular determinants of host specialization, the species *Xanthomonas axonopodis* [32]. Indeed, within this bacterial species, numerous pathovars displaying various plant host ranges have been defined [32]–[35]. Furthermore, it is worth noting that recent phylogenetic studies performed on strains belonging to *X. axonopodis* demonstrated that some pathovars did not form monophyletic groups [36], [37]. Some strains being phylogenetically very close may belong to different pathovars, whereas some strains belonging to the same pathovar may be distant phylogenetically. For example, the pathovar *phaseoli*, that groups all the strains pathogenic on bean, comprises four distinct genetic lineages [24]. Strains belonging to the genetic lineages 2 and 3 of *X. axonopodis* pv. *phaseoli* are phylogenetically closer to strains pathogenic on citrus or cotton, than strains belonging to the genetic lineage 1 of the pathovar *phaseoli* [24]. This suggests that in *X. axonopodis*, host specialization results from phylogeny-independent factors.

Interestingly, *Xanthomonas* species comprise pathovars that have different tissue specificities. For example, the species *X. oryzae* and *X. campestris* include pathovars that invade their hosts through the vascular system ( = vascular pathogens) and pathovars that colonize the intercellular spaces of the parenchyma tissue ( = non-vascular pathogens) [38], [39]. Like *X. oryzae* and *X. campestris*, *X. axonopodis* also comprises vascular and non-vascular pathovars [40]–[56]. Tissue specificity is also reported to be targeted by T3Es [57].

In this study, we assayed for the presence of 35 T3Es in a set of 132 strains of the species *X. axonopodis*, representative of 18 different pathovars. We then tested for associations between pathovars and the repertoire of T3Es of the tested strains, to provide data challenging a “repertoire for repertoire” hypothesis that may explain host and tissue specificity.

## Results

Presence or absence of 35 T3Es in strains used in this work was achieved by using specific primers for PCR amplification, as well as by dot blot hybridization. Both methods were used simultaneously to obtain complementary results: hybridization tells whether strains contain orthologs of the probed T3E gene, whereas PCR approach provides clues on whether genetic rearrangements may have occurred in the target sequence.

Phylogenetic position of strains used in this work was obtained by sequencing the housekeeping gene *rpoD*. Comparison of dendrograms obtained using the *rpoD* phylogenetic data and the dendrogram obtained based on presence or absence of T3Es documents the involvement of T3E repertoires in host specialisation.

### The *X. axonopodis* species comprises monophyletic and polyphyletic pathovars

**Figure 1:**
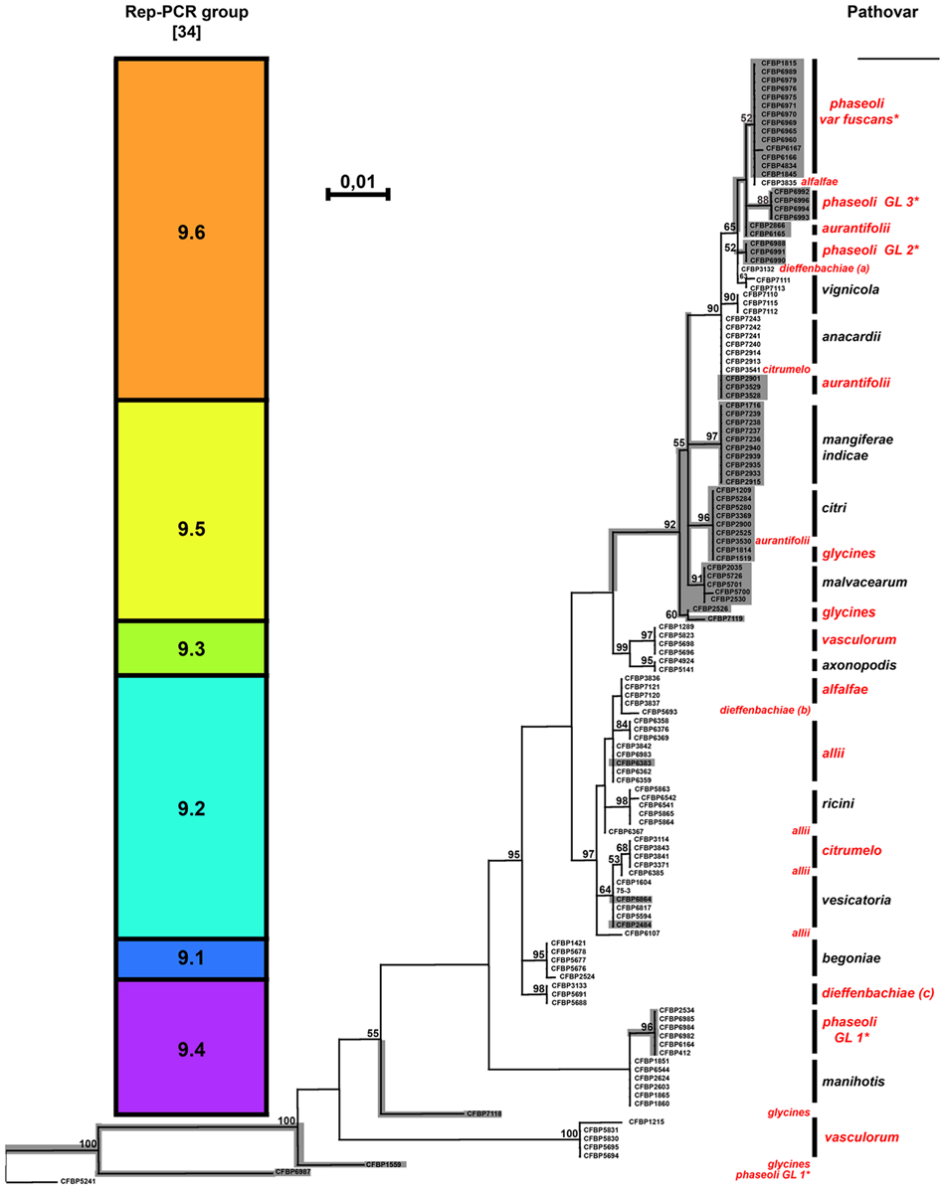
Phylogeny of 18 pathovars of *Xanthomonas axonopodis* based on *rpoD* gene sequences. The phylogenetic tree was constructed using the method of maximum likelihood. Confidence on nodes was tested with 1000 bootstrap replicates. Bootstrap values under 50 are not reported. Bar, 0.01 substitution per site. The tree is rooted with the *rpoD* gene sequence of strain CFBP 5241 of *Xanthomonas campestris* pv. *campestris*. The tree constructed based on *rpoD* sequences is congruent with previous grouping based on Rep-PCR profiles by Rademaker [34]. Polyphyletic pathovars are reported in red, whereas monophyletic are reported in black. *GL1, GL2, GL3, and var. *fuscans* correspond to the 4 genetic lineages previously described in the pv. *phaseoli* [24]. The dendrogram also displays the evolutionnary history of the T3E *xac3090* as inferred from the parsimony method implemented in Mesquite [89]. Occurence of *xac3090* is indicated on the tree by gray branches. For example, *xac3090* is present in all the strains of pv. *glycines*. The parsimony analysis indicates that this T3E appeared several independent times in strains of *X. axonopodis* pv. *glycines*, as well as in other parts of the tree.

In order to test whether the phylogeny could explain the distribution of the strains of our collection (Table 1) among the 18 pathovars of *X. axonopodis*, we sequenced *rpoD*, one of the housekeeping genes commonly used in multilocus sequence analysis and typing (MLSA and MLST) studies [37], [58]–[61]. Phylogenetic trees based in *rpoD* sequences were constructed using the method of Maximum Likelihood. Akaike information criterion used in Modeltest [62] selected the GTR+I+G model with the following parameters: f(A) = 0.1368, f(C) = 0.3256, f(G) = 0.3110; rate matrix R(A–C) = 1.9312, R(A–G) = 12.5584, R(A–T) = 0.4209, R(C–G) = 1.2602, R(C–T) = 2.6835, R(G–T) = 1; proportion of invariable sites (I) = 0.5065 and Gamma distribution shape parameter (G) = 0.7388. The Maximum Likelihood tree, rooted with the orthologous *rpoD* sequences from *X. campestris* pv. *campestris* strain CFBP5241, is presented in Figure 1. A very similar tree was also obtained with sequences of four housekeeping genes originating from about forty strains belonging to diverse pathovars of *X. axonopodis* [36], [37]. The tree constructed based on *rpoD* sequences matched well with the 6 rep-PCR clusters (from 9.1 to 9.6) defined within the *X. axonopodis* species (Figure 1) [34], [35]. Moreover, high bootstrap values indicated that this clustering was well supported and that the tree was robust (Figure 1). It is worth observing that, depending on the *X. axonopodis* pathovars, strains were grouped together or were distributed into phylogenetically unrelated groups (Figure 1). From this phylogenetic analysis, pathovars *anacardii*, *axonopodis*, *begoniae*, *citri*, *mangiferaeindicae*, *malvacearum*, *manihotis*, *ricini*, *vesicatoria* and *vignicola* can be considered as monophyletic whereas pathovars *alfalfae*, *allii, aurantifolii*, *citrumelo*, *dieffenbachiae*, *glycines*, *phaseoli* and *vasculorum* can be considered as polyphyletic.

**Table 1:** Bacterial strains used in this study.

Species/Pathovars	Strains	Other collections	Host of isolation	Geographic origin	Year of isolation
*X. axonopodis* pv.					
*alfalfae*	CFBP 3835	ICMP 3376	*Medicago sativa*	Australia	1972
*alfalfae*	CFBP 3836	ICMP 5718, LMG 497, NCPPB 2062	*Medicago sativa*	Sudan	NA
*alfalfae*	CFBP 3837	ICMP 9115	*Medicago sativa*	United States	1965
*alfalfae*	CFBP 7120	NCPPB 1821	*Medicago sativa*	Japan	1962
*alfalfae*	CFBP 7121	NCPPB 480	*Medicago sativa*	India	NA
*allii*	CFBP 6107	MAFF 311173	*Allium fistulosum*	Japan	1998
*allii*	CFBP 6369		*Allium cepa*	Réunion Island	1996
*allii*	CFBP 6358		*Allium sativum*	Réunion Island	1994
*allii*	CFBP 6359	LMG 580, ICPB XC177	*Allium cepa*	United States	1980
*allii*	CFBP 6362	IBSBF 594, ICMP 9278	*Allium cepa*	Brazil	1986
*allii*	CFBP 6364		*Allium sativum*	Cuba	1986
*allii*	CFBP 6367		*Allium cepa*	Barbados	NA
*allii*	CFBP 6376		*Allium cepa*	Mauritius	1997
*allii*	CFBP 6383		*Allium cepa*	United States	NA
*allii*	CFBP 6385		*Allium cepa*	South Africa	NA
*anacardii*	CFBP 2914	ICMP 4087	*Mangifera indica*	Brazil	NA
*anacardii*	CFBP 2913	ICMP 4088	*Mangifera indica*	Brazil	NA
*anacardii*	CFBP 7240	JY542	*Anacardium occidentale*	Brazil	2001
*anacardii*	CFBP 7241	LA099	*Anacardium occidentale*	Brazil	2004
*anacardii*	CFBP7242	LA100	*Anacardium occidentale*	Brazil	2004
*anacardii*	CFBP 7243	LA102	*Anacardium occidentale*	Brazil	2004
*aurantifolii*	CFBP 3528		*Citrus limon*	Argentina	1988
*aurantifolii*	CFBP 3529		*Citrus limon*	Uruguay	1983
*aurantifolii*	CFBP 3530		*Citrus limon*	Uruguay	1984
*aurantifolii*	CFBP 2901		*Citrus limon*	Argentina	NA
*aurantifolii*	CFBP 2866	NCPPB 3233	*Citrus aurantiifolia*	Brazil	1982
*axonopodis*	CFBP 4924	LMG 539, ICMP 698, NCPPB 2375	*Axonopus scoparius*	Colombia	1949
*axonopodis*	CFBP 5141	LMG 538, ICMP 50, NCPPB 457	*Axonopus scoparius*	Colombia	1949
*begoniae*	CFBP 2524	ICMP 194, LMG 7303, NCPPB 1926	*Begonia sp.*	New Zealand	1962
*begoniae*	CFBP 5677		*Begonia pendula*	France	1991
*begoniae*	CFBP 1421		*Begonia sp.*	France	NA
*begoniae*	CFBP 5676		*Begonia rugosa*	Antilles	1988
*begoniae*	CFBP 5678		*Begonia eliator*	Germany	1994
*citri*	CFBP 2525	NCPPB 409, ICMP 24, LMG 682	*Citrus limon*	New Zealand	1956
*citri*	CFBP 3369	LMG 9322, ATCC 49118.	*Citrus aurantifolia*	United States	1989
*citri*	CFBP 1209	NCPPB 1472	*Citrus grandis*	Hong Kong	1963
*citri*	CFBP 1814		*Citrus sp.*	Réunion Island	1978
*citri*	CFBP 5280		*Citrus hystrix*	Thailand	1998
*citri*	CFBP 5284		*Citrus sp.*	Malaysia	1999
*citri*	CFBP 2900		*Citrus sp.*	Japan	NA
*citri*		306	NA	NA	NA
*citrumelo*	CFBP 3371	LMG 9325, ICPB 10483	NA	NA	1989
*citrumelo*	CFBP 3541		*Citrus aurantiifolia*	Mexico	NA
*citrumelo*	CFBP 3841	LMG 9160.	*Poncirus trifoliata x citrus sinensis*	United States	NA
*citrumelo*	CFBP 3842	LMG 9167.	*Poncirus trifoliata x citrus paradisi*	United States	NA
*citrumelo*	CFBP 3843	LMG 9172.	*Citrus paradisi*	United States	NA
*citrumelo*	CFBP 3114		*Citrumelo cv. Swingle*	United States	1984
*dieffenbachiae*	CFBP 3133	NCPPB 1833, ICMP 5727, LMG 695	*Anthurium sp.*	Brazil	1965
*dieffenbachiae*	CFBP 3132	NCPPB 985, LMG 7399	*Diffenbachia sp.*	United States	1950
*dieffenbachiae*	CFBP 5688		*Anthurium andreanum*	Venezuela	NA
*dieffenbachiae*	CFBP 5693		*Philodendron scandens*	United States	NA
*dieffenbachiae*	CFBP 5691		*Anthurium sp.*	Mauritius	NA
*glycines*	CFBP 1519	NCPPB 1717.	*Glycine hispida*	Zimbabwe	1962
*glycines*	CFBP 2526	NCPPB 554, ICMP 5732, LMG 712.	*Glycine hispida*	Sudan	1956
*glycines*	CFBP 7119	NCPPB 3658	*Glycine max*	Brazil	1981
*glycines*	CFBP 7118	NCPPB 1716	*Glycine javanica*	Zambia	1963
*glycines*	CFBP 1559		*Glycine hispida*	France	1974
*malvacearum*	CFBP 2035		*Gossypium hirsutum*	Argentina	1981
*malvacearum*	CFBP 2530	NCPPB 633, ICMP 5739, LMG 761.	*Gossypium hirsutum*	Sudan	1958
*malvacearum*	CFBP 5700		*Gossypium hirsutum*	Senegal	1990
*malvacearum*	CFBP 5701		*Gossypium hirsutum*	Madagascar	1990
*malvacearum*	CFBP 5726		*Gossypium barbadense*	Sudan	1991
*mangiferaeindicae*	CFBP 1716	ICMP 5740, LMG 941, NCPPB 490	*Mangifera indica*	India	1957
*mangiferaeindicae*	CFBP 2933		*Mangifera indica*	Réunion Island	1981
*mangiferaeindicae*	CFBP 7236	JN576	*Mangifera indica*	Japan	1993
*mangiferaeindicae*	CFBP 2915	NCPPB 2438	*Mangifera indica*	South Africa	1971
*mangiferaeindicae*	CFBP 2935		*Mangifera indica*	Australia	1978
*mangiferaeindicae*	CFBP 2939		*Schinus terebenthifolius*	Réunion Island	1987
*mangiferaeindicae*	CFBP 2940		*Schinus terebenthifolius*	Réunion Island	1987
*mangiferaeindicae*	CFBP 7238	JP742	*Schinus terebenthifolius*	Réunion Island	1994
*mangiferaeindicae*	CFBP 7239	JP757	*Schinus terebenthifolius*	Réunion Island	1994
*mangiferaeindicae*	CFBP 7237	JP740	*Schinus terebenthifolius*	Réunion Island	1994
*manihotis*	CFBP 1860		*Manihot esculenta*	Nigeria	1978
*manihotis*	CFBP 2603	NCPPB 2443.	*Manihot esculenta*	Colombia	1972
*manihotis*	CFBP 1851	CIAT111	*Manihot esculenta*	United States	NA
*manihotis*	CFBP 2624		*Manihot esculenta*	Réunion Island	1986
*manihotis*	CFBP 6544		*Manihot esculenta*	Brazil	1992
*manihotis*	CFBP 1865		*Manihot esculenta*	Congo	1977
*phaseoli* var *fuscans*	CFBP 1815		*Phaseolus sp.*	Greece	1978
*phaseoli* var *fuscans*	CFBP 4834		*Phaseolus vulgaris*	France	1998
*phaseoli* var *fuscans*	CFBP 6165	LMG 826, ICMP 239, NCPPB 381.	*Phaseolus vulgaris*	Canada	1957
*phaseoli* var *fuscans*	CFBP 6167	LMG 7511, ICMP 242.	*Phaseolus sp.*	United States	1964
*phaseoli* var *fuscans*	CFBP 6969		*Phaseolus vulgaris*	Tanzania	2001
*phaseoli* var *fuscans*	CFBP 6166	LMG 837, NCPPB 1654.	*Phaseolus vulgaris*	South Africa	1963
*phaseoli* var *fuscans*	CFBP 6965		*Phaseolus vulgaris*	NA	NA
*phaseoli* var *fuscans*	CFBP 6975		*Phaseolus sp.*	France	1994
*phaseoli* var *fuscans*	CFBP 6976		*Phaseolus sp.*	Switzerland	1994
*phaseoli* var *fuscans*	CFBP 6979		*Phaseolus vulgaris*	Tanzania	2001
*phaseoli* var *fuscans*	CFBP 1845		*Phaseolus sp.*	Greece	1978
*phaseoli* var *fuscans*	CFBP 6960		*Phaseolus vulgaris*	Réunion Island	2000
*phaseoli* var *fuscans*	CFBP 6970		*Phaseolus sp.*	United States	1990
*phaseoli* var *fuscans*	CFBP 6971		*Phaseolus sp.*	Tanzania	1992
*phaseoli* GL1	CFBP 2534	ATCC 9563, NCPPB 3035, ICMP 5834	*Phaseolus vulgaris*	United States	NA
*phaseoli* GL1	CFBP 6164	LMG 8014, NCPPB 1811.	*Phaseolus vulgaris*	Romania	1966
*phaseoli* GL1	CFBP 6987		*Phaseolus vulgaris*	Tanzania	NA
*phaseoli* GL1	CFBP 6984		*Phaseolus vulgaris*	Réunion Island	2000
*phaseoli* GL1	CFBP 6982		*Phaseolus vulgaris*	Réunion Island	2000
*phaseoli* GL1	CFBP 412		*Phaseolus vulgaris*	United States	NA
*phaseoli* GL1	CFBP 6983		*Phaseolus vulgaris*	Réunion Island	2000
*phaseoli* GL1	CFBP 6985		*Phaseolus vulgaris*	Réunion Island	2000
*phaseoli* GL2	CFBP 6989		*Phaseolus vulgaris*	Réunion Island	2000
*phaseoli* GL2	CFBP 6990		*Phaseolus vulgaris*	Réunion Island	2000
*phaseoli* GL2	CFBP 6991		*Phaseolus vulgaris*	Réunion Island	2000
*phaseoli* GL2	CFBP 6988		*Phaseolus vulgaris*	Réunion Island	2000
*phaseoli* GL3	CFBP 6992		*Phaseolus vulgaris*	Réunion Island	2000
*phaseoli* GL3	CFBP 6994		*Phaseolus vulgaris*	Tanzania	1990
*phaseoli* GL3	CFBP 6996		*Phaseolus vulgaris*	Réunion Island	2000
*phaseoli* GL3	CFBP 6993		*Phaseolus vulgaris*	Réunion Island	2000
*ricini*	CFBP 5863	IBSBF 313.	*Ricinus communis*	Brazil	1981
*ricini*	CFBP 5864	IBSBF 1191.	*Ricinus communis*	Brazil	1995
*ricini*	CFBP 5865	IBSBF 1192	*Ricinus communis*	Brazil	1995
*ricini*	CFBP 6541		*Ricinus communis*	Brazil	1981
*ricini*	CFBP 6542		*Ricinus communis*	Brazil	1985
*vasculorum*	CFBP 1215		*Saccharum officinarum*	Kenya	NA
*vasculorum*	CFBP 5694		*Zea mays*	Réunion Island	NA
*vasculorum*	CFBP 5695		*Tripsacum laxum*	Réunion Island	NA
*vasculorum*	CFBP 5696		*Thysanolena maxima*	Réunion Island	NA
*vasculorum*	CFBP 5698		*Saccharum officinarum*	Trinidad	NA
*vasculorum*	CFBP 5823	LMG 901, ICMP 5757, NCPPB 796.	*Saccharum officinarum*	Mauritius	1979
*vasculorum*	CFBP 1289		*Saccharum officinarum*	Réunion Island	1970
*vesicatoria*	CFBP 1604		*Capsicum annuum*	Guadeloupe.	NA
*vesicatoria*	CFBP 5594		*Lycopersicon esculentum*	Guadeloupe	1993
*vesicatoria*	CFBP 5618	Xcv. 85-10	*Capsicum annuum*	United States	NA
*vesicatoria*		75-3	*Lycopersicon esculentum*	NA	NA
*vesicatoria*	CFBP 2484		*Lycopersicon esculentum*	Guadeloupe	1980
*vesicatoria*	CFBP 6864		*Capsicum frutescens*	United States	1947
*vesicatoria*	CFBP 6817		NA	Thailand	1997
*vignicola*	CFBP 7110	LMG 831, NCPPB 638	*Vigna unguiculata*	Zimbabwe	NA
*vignicola*	CFBP 7111	NCPPB 1633	*Vigna sinensis*	United States	1942
*vignicola*	CFBP 7112	LMG 8752, NCPPB 1838	*Vigna unguiculata*	United States	1942
*vignicola*	CFBP 7113	LMG 840, NCPPB 2061	*Vigna unguiculata*	Sudan	1966
*vignicola*	CFBP 7115	NCPPB 3187	*Vigna sinensis*	Brazil	1978
*X. campestris* pv. *campestris*	CFBP 5241	LMG 568, ICMP 13, NCPPB 528	*Brassica oleracea*	United Kingdom	1957
*X. campestris* pv. *campestris*	CFBP 6650	LMG 8004, NCPPB 1145	Brassica oleracea	United Kingdom	1958
*X. oryzae* pv. *oryzae*	CFBP 7088	KACC10331	*Oryza sativa*	Korea	
*X. oryzae* pv. *oryzicola*	CFBP 7109	BLS256	*Oryza sativa*	Philippines	1984
*X. albilineans*	CFBP 7063		*Saccharum spp.*	Guadeloupe.	2003
*Escherichia coli*		DH5α			

The *rpoD*-based tree was sufficient to highlight the existence of different genetic lineages within a pathovar. As an example, the four genetic lineages of the pathovar *phaseoli* that have been determined by Amplified Fragment Length Polymorphism (AFLP) [24, our unpublished data], were supported by the *rpoD* sequence analysis (Figure 1). Our *rpoD*-based tree confirmed that strains belonging to the genetic lineage 1 of the pathovar *phaseoli* are phylogenetically distant from strains belonging to the three other genetic lineages. *X. axonopodis* pv. *phaseoli* strain CFBP6987 diverged from all other strains belonging to this pathovar, which also supports our previous AFLP analyses [our unpublished data]. Concerning strains of the pathovar *dieffenbachiae*, it is interesting to notice that *rpoD* sequences analysis gather strains into three phylogenetic groups according to their host of isolation (*Anthurium* sp., *Dieffenbachia* sp. and *Philodendron* sp.) (Figure 1). Another striking observation was that some strains belonging to different pathovars (*aurantifolii*, *citri* and *glycines* in a first case and *anacardii*, *aurantifolii* and *citrumelo* in a second case) exhibited identical *rpoD* sequences (Figure 1).

### T3E repertoires of *X. axonopodis* strains combine ubiquitous and variable effectors

**Figure 2:**
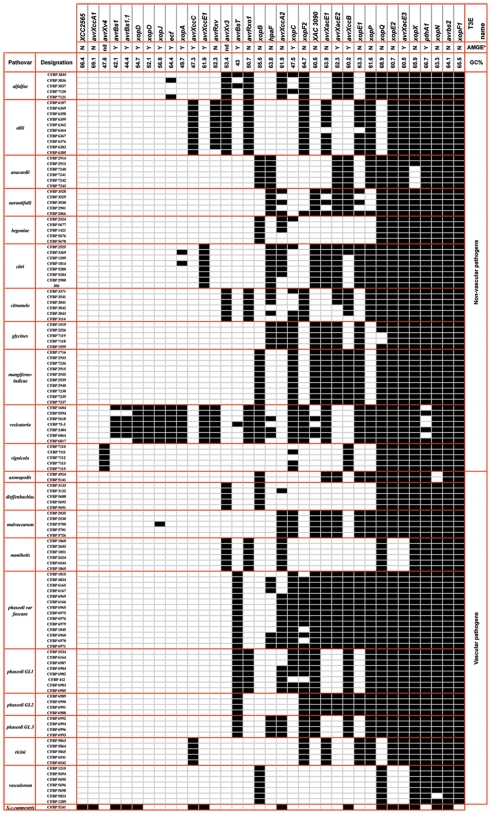
Distribution of T3E genes among strains belonging to 18 pathovars of *Xanthomonas axonopodis*. In this figure, the presence or the absence of an ortholog of each selected T3E gene was determined by dot-blot hybridizations. Black squares represent presence of the corresponding gene, whereas white squares represent absence of sequence similar to the probe used. In the latter case, gene may be absent or its sequence is too divergent to be detected. The GC% of each T3E gene is indicated on the basis of sequences of the orthologs found in the databases. *AMGE indicates whether the considered gene was reported to be Associated to Mobile Genetic Elements in *Xanthomonas* strains whose genome was sequenced (Y:Yes; N: No) [8], [64].

Before investigating the T3E gene distribution among our collection of strains, we checked that all strains of our collection have a T3SS of the Hrp2 family that is usually present in xanthomonads [8], [9], [63]–[66]. This analysis, performed by using specific PCR primers [55], revealed that all strains of our collection carry this T3SS (data not shown). Then, the distribution study of the 35 selected T3E genes among the *X. axonopodis* strains was studied and the results are presented in Figure 2 and in Table S1. When we did not detect the presence of a T3E gene in a given strain, we considered that this gene is absent or too divergent to be detected. Indeed, our approach cannot completely rule out the fact that some T3E genes may have been subjected to diversifying selection which resulted in a sufficient divergence sequence to avoid detection through dot-blot hybridization.

Our results clearly revealed that T3E repertoires contained two categories of genes. Some genes showed a broad distribution among strains whereas the remaining ones displayed a variable distribution. The first class comprised T3E genes (*xopF1*, *avrBs2*, *xopN*, *pthA1*, *xopX*, *xopQ*, *avrXacE3* and *xopE2*) that were present in at least 87% of the strains tested. These genes will be referred to as ubiquitous T3Es. We could then consider ubiquitous T3Es as the core suite of T3E genes for strains of *X. axonopodis*. The other T3Es, which distribution were not as broad as the ubiquitous genes, constituted a second class of genes: for instance *xopP* was detected in 67% of the strains, but 10 other genes were present in less than 10% of the *X. axonopodis* strains tested. Two of them, *avrXccA1* and *XCC2565*, were not detected in any of the strains, although they were found in *X. campestris* pv. *campestris* strain CFBP5241. This second class of genes will be referred to as variable T3Es. We could then consider variable T3Es as the variable suite of T3E genes for *X. axonopodis* strains. Interestingly, all ubiquitous T3Es have a G+C content (∼65%) similar to the average value of total DNA for *X. axonopodis* strains [8], [32], [64] whereas the majority of variable T3E genes have a G+C content considerably lower (until 42.1%) (Figure 2). Another point of interest is the association of the selected T3Es with mobile elements when looking at the sequenced genomes of *Xanthomonas* strains [8], [64]. Indeed, the majority of the variable T3E genes appeared to be associated with IS elements, contrary to what is observed for ubiquitous T3E genes (Figure 2). Moreover, ubiquitous T3E genes are flanked by orthologous genes in *X. axonopodis* genomes, in contrast to what is seen for the majority of variable T3E genes [8], [64].

### Different pathovars have different T3E repertoires. Some diversity in T3E repertoires may be observed within some pathovars

T3E repertoires were highly variable between *X. axonopodis* strains, both in terms of T3E present and of size of the repertoires (Figure 2). Our results showed that repertoires of T3Es were different between strains belonging to different pathovars.

When looking at the size of T3E repertoires, we observed a large variability. Strains of the pathovar *vasculorum* harboured the smallest T3E repertoire (6 or 7 of the 35 selected T3E genes depending on the strains) whereas strains of pathovar *vesicatoria* exhibited the largest T3E repertoire (from 22 to 26 of the 35 selected T3E genes depending on the strains). Regarding strains of the 16 remaining pathovars, their T3E repertoires were composed of 10 to 20 of the 35 selected T3E genes.

Within most pathovars, repertoires of T3Es were conserved. Indeed, we observed identical or almost identical (only one T3E gene in one strain differs) T3E repertoires from strains of the monophyletic pathovars *anacardii*, *axonopodis*, *malvacearum*, *manihotis*, *mangiferaeindicae*, *ricini* and *vignicola* (Figure 2). We also observed almost identical T3E repertoires (only one T3E gene in one or two strains differs) from strains belonging to the polyphyletic pathovars *dieffenbachiae*, *glycines* and *vasculorum*. For example, strain CFBP3132 of the pathovar *dieffenbachiae* carries one more gene (*avrXccA2*) than other strains of this pathovar (Figure 2).

When a significant variation in T3E repertoires occurred between strains of the same pathovar, the observed variation can be linked to the reported genetic diversity within this pathovar. For example, the four genetic lineages, that were defined in the pathovar *phaseoli* [24], possess T3E repertoires that are similar but not identical (Figure 2). Some variations in T3E repertoires within genetic lineages of the pathovar *phaseoli* were noticed as well, but differences are smaller among strains belonging to the same genetic lineage than among strains belonging to different genetic lineage. In other cases, the variation observed within a pathovar can be linked to the host of isolation. For example, among strains of the pathovar *anacardii*, T3E repertoires are almost identical, but strains isolated from *Mangifera indica* (CFBP2913 and CFBP2914) carry one more T3E gene (*xopX*) than strains isolated from *Anacardium occidentale* (CFBP7240, CFBP7241, CFBP7242, 7243) (Figure 2). A similar observation can be made for pathovar *glycines* strains isolated from *Glycine hispida* (CFBP1519, CFBP1559 and CFBP2526) which carry one more T3E gene (*xopQ*) than strains isolated from *Glycine javanica* or *Glycine max* (CFBP7118 and CFBP7119) (Figure 2).

### Distance between studied repertoires highlights a correspondence between T3E repertoires and pathovars of *X. axonopodis*

**Figure 3:**
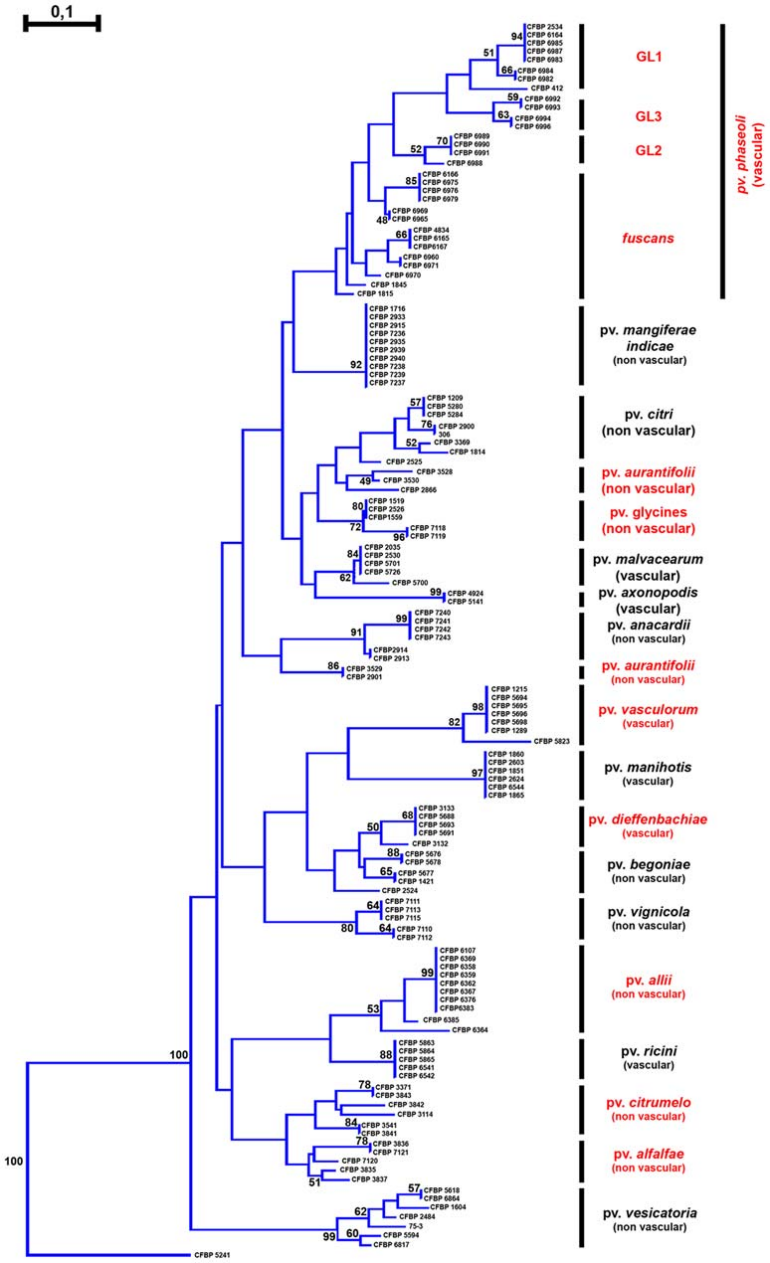
Dendrogram constructed based on results of presence/absence of T3Es in 18 pathovars of *Xanthomonas axonopodis*. The tree was constructed with the Neighbor Joining method using Jaccard distances and rooted with the strain CFBP 5241 of *Xanthomonas campestris* pv. *campestris*. Confidence on nodes was tested with 1000 bootstrap replicates. Bootstrap values under 50 are not reported. Polyphyletic pathovars are reported in red, whereas monophyletic are reported in black. *GL1, GL2, GL3, and *fuscans* refer to the 4 genetic lineages previously described in *X. axonopodis* pv. *phaseoli* [24].

To test the hypothesis of a correspondence between repertoires of T3Es and pathovars, we constructed a dendrogram from a matrice summarizing presence/absence of the T3Es for each of the 132 selected strains. Strikingly, this dendrogram grouped the tested strains according to their pathovar, and these groupings were supported by high bootstrap values (Figure 3). However, one exception was observed since strains of the pathovar *aurantifolii* were distributed in two distinct groups. Such result was particularly interesting when looking at the case of pathovars that appeared polyphyletic based on our *rpoD* sequence analysis (see above). For example, strains of the pathovars *citrumelo*, *dieffenbachiae, glycines*, and *vasculorum*, which have been split into phylogenetic distinct groups (Figure 1), clustered together based on T3E repertoires (Figure 3). Regarding the pathovar *phaseoli*, the four genetic lineages appeared tightly related when looking at the T3E repertoires whereas one genetic lineage was phylogenetically distant from the three other ones (Figures 1 and 3). The same observation could be made for the pathovar *phaseoli* strain CFBP6987, that was evolutionary divergent based on *rpoD* sequencing (Figure 1) but indistinguishable from other strains of the genetic lineage 1 of the pathovar *phaseoli* based on T3E repertoires (Figure 3).

Therefore, our results revealed some correlations between repertoires of T3Es and pathovars. This suggests that T3E repertoires might promote the pathogenicity of strains that are phylogenetically distinct on the same host plants. Thus, T3E repertoires represent candidate determinants of the pathological adaptation of the *X. axonopodis* strains on their hosts.

Furthermore, some T3Es may allow to discrimate between pathovars. Indeed, among the T3Es we tested, some appear specific of certain pathovars. For example, *xopD* and *xopO* were specific of the pathovar *vesicatoria*. Some T3E genes also allowed discrimination between different genetic lineages of polyphyletic pathovars. For example, *avrRxo1* allowed discrimination between genetic lineage 1 of the pathovar *phaseoli* and the other genetic lineages. Genetic lineages 2 and *fuscans* may be discriminated from the two other genetic lineages of the pathovar *phaseoli* by the presence of *avrXacE1* and *avrXacE2*.

When one considered the tissue specificity of the *X. axonopodis* strains, our results presented in Figures 2 and 3 showed no clear delineation between vascular and non-vascular pathogens. We did not detect T3E genes that allow distinction between vascular and non-vascular pathovars. No apparent correlation was observed between T3E repertoires and tissue specificity of the tested strains in contrast to our results between T3E repertoires and host specificity. Nevertheless, in Figure 3 we noted that certain vascular pathogens, such as pathovars *vasculorum* and *manihotis*, or certain non-vascular pathogens, such as pathovars *citrumelo* and *alfalfae*, appeared closely related in the dendrogram but these groupings were not supported by high bootstrap values.

### Association between T3E genes within repertoires

The notion that a T3E repertoire enables a pathological convergence on a particular host implies that it is the coordinated action of several T3Es rather than the action of one unique T3E that matters. Thus, we wanted to estimate potential associations between T3Es, which may provide clues on potential functional synergies or redundancies between pairs of T3Es.

**Figure 4:**
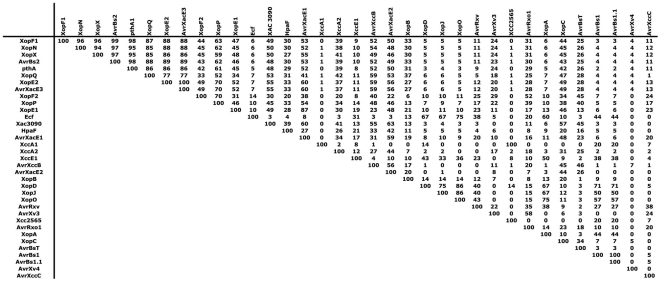
Frequency of association between T3Es in *Xanthomonas axonopodis*. Numbers represent the frequency of cases when both T3Es in the pair are present in the same strains.

Therefore, we calculated for each pair of T3E gene a frequency of association, and we considered only cases where both T3E genes in a pair are present in the tested strains (Figure 4). When we found high frequencies of association between T3E genes in *X. axonopodis*, we revealed that either these genes are genetically-linked or -unlinked based on the genome sequence of the *X. axonopodis* pv. *vesicatoria* strain CFBP5618 ( = strain 85-10) [64]. The first case is illustrated by *avrBs1* and *avrBs1.1* that are genetically-linked and showed 100% of association (Figure 4). Interestingly, the observed genetic linkage of *xopN* and *xopF2* in *X. axonopodis* pv. *vesicatoria* strain CFBP5618 [64] does not seem to be conserved in all *X. axonopodis* strains since both genes, when present, did not exhibit 100% of association but 45% of association (Figure 4). The second case is illustrated for instance by *avrBsT* and *xccB* (46% of association) or by *avrRxv* and *xopJ* (43% of association) (Figure 4) that are genetically-unlinked. This point is particularly important when one consider the functional families of T3Es and then functional redundancy between T3Es. For instance, *avrBsT*, *xccB*, *avrRxv* and *xopJ* belong to the same functional family, namely the YopJ/AvrRxv family of cysteine proteases (Table S2) [67], [68]. Interestingly, we also found high frequencies of association between genetically-unlinked T3Es belonging to the HopX/AvrPphE family (Table S2) [8], [68]. For example, we found that *avrXacE1* was highly associated with *xopE1* (87%), *xopE2* (60%), *avrXacE3* (60%) and *avrXacE2* (59%) (Figure 4). Regarding genetically-unlinked *hpaF* and *xac3090* which encode T3Es belonging to the PopC family (Table S2) [8], it appeared that both genes showed 39% of association (Figure 4). In contrast, genetically-unlinked *xopX* and *ecf* that encode T3Es belonging to the HopAE1 family (Table S2) [68] exhibited a very low frequency of association (only 6%) in *X. axonopodis* strains (Figure 4).

### Few DNA rearrangements are identified within T3E genes

We tried to detect the presence of the 35 selected T3E genes in our large *X. axonopodis* strains collection by both PCR and dot-blot hybridization methods. Interestingly, we observed that some PCR products were clearly different in size from the expected PCR products. Indeed, 22 PCR products were larger and one was smaller as compared with PCR products of the reference strains, suggesting insertion or deletion of DNA sequences within some T3E genes. To further characterize the DNA rearrangements within these T3E genes, we sequenced the PCR fragments generated from these genes. The sequence analyses revealed three types of DNA rearrangements: deletion, tandem duplication and insertions of IS element (Table 2). We identified one in-frame deletion of 384 bp in the *xopF2* gene of *X. axonopodis* pv. *aurantifolii* strain CFBP2866, the only strain of this pathovar carrying this gene (Table 2 and Figure 2). One perfect tandem duplication of 90 bp in size was identified in *xopD* of the *X. axonopodis* pv. *vesicatoria* strain CFBP6817 (Table 2).

**Table 2:** DNA rearrangements found in T3E genes from *Xanthomonas axonopodis* strains.

T3E genes	Pathovars	Strains	DNA rearrangements			
			Type^a^	Size (bp)	Position^b^	IS direct repeat sequence generated by IS insertion and duplicated or deleted sequence in T3E genes plus flanking sequence^c^
*avrXv3*	*alfalfae*	CFBP3835, CFBP3836, CFBP3837, CFBP7120, CFBP7121	IS*1595* (IS*1595* family, IS*1595* group)	1072	513	AAGATTCA
*xopO*	*vesicatoria*	CFBP6817	IS*Xca2* (IS*3* family, IS*407* group)	1200	33	CCAT
*ecf*	*alfalfae*	CFBP3836	IS*Xca2* (IS*3* family, IS*407* group)	1200	1423	CGTT
*avrRxo1*	*allii*	CFBP6369, CFBP6358	IS*Xca2* (IS*3* family, IS*407* group)	1200	770	GACG
*avrRxo1*	*allii*	CFBP6107	IS*1389* (IS*3* family, IS*407* group)	1207	411	GTCC
*avrXacE2*	*alfalfae*	CFBP3836, CFBP3837	IS*Xca2* (IS*3* family, IS*407* group)	1200	558	AGGG
*xopC*	*citrumelo*	CFBP3114, CFBP3371, CFBP3843	IS*1404* (IS*3* family, IS*407* group)	1203	2034	GCGA
*xopC*	*mangiferae-indicae*	CFBP2939, CFBP2940, JP740, JP742, JP757	IS*1479* (IS*5* family, IS*5* group)	1154	1091	CTAG
*xopN*	*aurantifolii*	CFBP2866	IS*Xac2* (IS*3* family, IS*407* group)	1195	ND	
*xopF2*	*aurantifolii*	CFBP2866	Deletion	384	1030–1413	gccgt[ATCGTCCAGCACGGACTGACGCTGCGCGATTCCTGCCCGACGGTCATAGGCCTGTGCCGTGAGCTCGGCACGGGTCGGCGTACCGTTTTCTCCGGGACGCACGAAGCGCTTGCTTCCCTCAGGGGTGATTTCCAATAGGACCGGCGGCGGGTCCGGCACCAGGATCTTGGGATCGATCGCCTGGAAACGCGGCAGATTGGCGACGCGCGCGCGCCTGTCCATCGATGGAATCAACAGCGTGTCGCAGGCATGCGACCCCATTCCCGAGATCACCCCAGCCAGGGTCGCTGTACTCAATGGCTCGGAAGCGGGCGGGCGGTTGCTCTGGTAGGCAGTGGCTGCAGAAATGCCGAGCGGCGTCCGCAACGCGCCAACGAAAAACGA]tctga
*xopD*	*vesicatoria*	CFBP6817	Tandem duplication	90	527–616	tcccc**ATACTCCGGCGGGTTCTTCCTATTCGTCCCTGTTCCCGCCCACCCCTTCTGGCGGTTGGCCGCAGAACGCATCAGGTGAGTGGCATCCCG** ***ATACTCCGGCGGGTTCTTCCTATTCGTCCCTGTTCCCGCCCACCCCTTCTGGCGGTTGGCCGCAGAACGCATCAGGTGAGTGGCATCCCG***atact

a IS names, families and groups are given according to IS Finder database (http://www.is.biotoul.fr/is.html).

b Nucleotide numbers are given considering the nucleotides A of the start codon of T3E genes as 1.

c Targets for duplication are in bold capitals, resulting tandem duplication are in bold and in italic, and deleted regions are in capitals in square brackets. Nucleotides flanking target regions, tandem duplications and deleted regions are in lower case.

**Figure 5:**
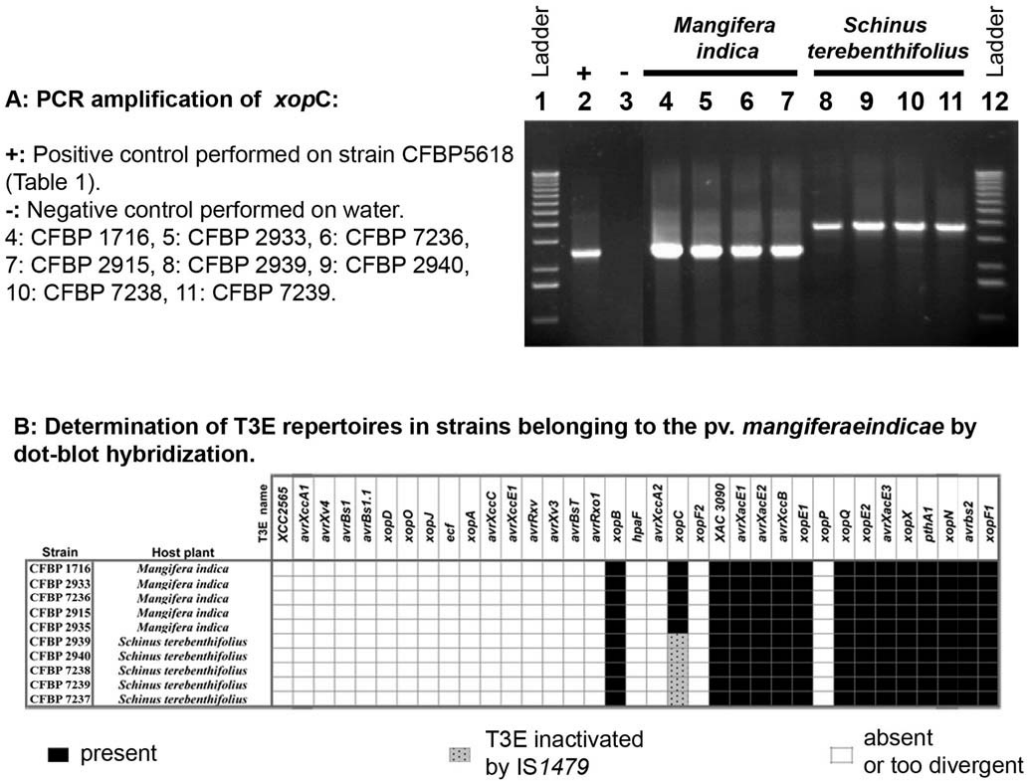
Among strains of pv. *mangiferaeindicae*, disruption of *xopC* by an IS element correlates with pathogenicity on *Schinus terebenthifolius*. Strains belonging to the pv. *mangiferaeindicae* are either pathogenic on *Mangifera indica* or on *Schinus terebenthifolius*. A: PCR experiments reveal the insertion of the IS*1479* element in all strains pathogenic on *Schinus terebenthifolius*. In contrast, strains pathogenic on *Mangifera indica* display a *xopC* gene that is not inactivated by IS*1479*. B: However, all strains display identical repertoires in dot-blot hybridization experiments. Such result suggests that inactivation of *xopC* may explain a pathogenicity switch between *Mangifera indica* and *Schinus terebenthifolius*.

Most of the DNA rearrangements (21/23) corresponds to insertions of IS elements. We found that seven T3E genes (*avrXv3*, *avrXacE2*, *avrRxo1*, *ecf*, *xopC*, *xopO*, *xopN*) from strains belonging to 6 pathovars of *X. axonopodis* were disrupted by 6 different IS elements (IS*1595*, IS*Xca2*, IS*Xac2*, IS*1389*, IS*1404* and IS*1479*). Interestingly, except IS*1479* and IS*1595*, these IS elements are closely related since they are classified within the single IS*3* family - IS*407* group (http://www-IS.biotoul.fr/is.html). The determination of the usual 4 bp DRs generated by insertions of the IS elements belonging to the IS*3* family-IS*407* group revealed no consensus sequence thus reflecting no insertion site specificity (Table 2). The determination of the location of IS element insertions (Table 2) showed that a T3E gene can be disrupted at the same position by the same IS element in all strains of the same pathovar (for example *avrXv3* disrupted by IS*1595* at position 513 in all pathovar *alfalfae* strains) or at different positions by different IS elements in different strains of the same pathovar (for instance *avrRxo1* disrupted by IS*Xca2* and IS*1389* at positions 770 and 411 respectively in strains CFBP6369 and CFBP6107 of the pathovar *allii*). We also observed that a T3E gene can be disrupted by different IS elements at different positions in strains belonging to different pathovars. This is the case of *xopC* that is disrupted by IS*1404* and IS*1479* in strains of pathovars *citrumelo* and *mangiferaeindicae* respectively. In this latter example, it is interesting to note that *xopC*, carried by pathovar *mangiferaeindicae* strains, is altered in strains isolated from *Schinus terebenthifolius* but not in those isolated from *Mangifera indica*. Since strains from both hosts of isolation exhibited identical T3E repertoires (Figures 2 and 5), this result might suggest that the alteration of this T3E gene might have a role in host adaptation for pathovar *mangiferaeindicae* strains.

To gain insight on sequence variation among orthologs of variable T3E, a subset of 120 sequences of variable T3Es was obtained. Genetic diversity thus observed was extremely reduced, and sequences obtained were almost identical to that of sequences of the functional orthologs found in the databases (data not shown). Only the sequence of *avrXccB* in strain CFBP1845 of the pathovar *phaseoli* displayed a premature stop codon (data not shown).

## Discussion

In this paper, we investigated the distribution of 35 T3Es among 132 strains belonging to 18 pathovars of the species *X. axonopodis* [32]. To our knowledge, this strain collection is the largest used in any other distribution study of virulence-associated genes in plant pathogenic bacteria. To provide the largest diversity, strains were chosen to represent the broad host range, wide geographic distribution, and genetic diversity of the species *X. axonopodis* [32], [34], [35]. In the course of this study, the phylogeny of the 132 selected strains was also constructed based on the sequence of the housekeeping gene *rpoD* to provide the frame necessary for the analysis of the results of our distribution study.

### T3E repertoires of *X. axonopodis* strains combine core and flexible gene sets that may play distinct roles in pathogenicity and may have evolved differently

It is important to note that our results supports previous observations made for *Pseudomonas syringae* [16], [69], [70] or *Ralstonia solanacearum* [12]. Indeed, we identified two classes of genes within T3E repertoires of *X. axonopodis* strains. The first class comprises 8 ubiquitous T3E genes (*avrBs2*, *xopN*, *xopF1*, *xopX*, *pthA1*,*xopE2*, *avrXacE3* and *xopQ*) whereas the second class contains the remaining T3E genes that are variable among strains. Then, one can consider that the first class represents the core T3E genes set and the second one the flexible T3 genes set of *X. axonopodis* strains. Both genes sets may play distinct roles in pathogenicity of the strains and may have evolved differently.

Regarding pathogenicity, the core T3E genes set could provide virulence functions of broad utility and then target defence components broadly conserved among a wide range of hosts [11], [70], [71]. Loss of these ubiquitous T3Es would lead to loss of fitness for the pathogen. Indeed, such hypothesis is supported by experimental data accumulated over two decades in diverse laboratories. For instance, mutations in *avrBs2*, *xopX*, *xopN* or members of the AvrBs3/PthA family were shown to alter fitness and pathogenicity of strains belonging to pathovars of *X. axonopodis* [72]–[77]. However, not fitting this picture is *xopF1* and *xopQ*, for which inactivation does not seem to alter pathogenicity of *X. axonopodis* pv. *vesicatoria* [74]. No data in the literature are available for both *xopE2* and *avrXacE3* genes. In contrast, the flexible T3E genes set could contribute to strategies specific to particular plant pathogen-host interactions and thus could account for host-specificity of plant pathogenic bacteria [11], [70], [71]. The role of variable T3Es would be then more subtle, and loss of such effectors may not be necessarily associated with a decrease of pathogenicity. For example, *avrBsT* is a variable effector as it is mainly found in strains belonging to the pathovar *phaseoli*. Inactivation of *avrBsT* does not seem to alter the pathogenicity of *X. axonopodis* pv. *phaseoli* [our unpublished data]. The same observations were made for *xopC*, *xopF2*, *xopJ*, *xopO* and *xopP* that appeared as variable T3E genes in our study [74], [78]. Altogether, these data, obtained from *Xanthomonas* strains, can be compared to what is known in *Pseudomonas syringae*. Indeed, mutations in T3Es of the conserved effector locus (CEL) usually alter pathogenicity [79]. Substantial experimental evidence is available for *hopPtoM*, *hopPtoN* and *avrE* in *Pseudomonas syringae* [27], [28], [80], as well as for *dspA/E* in *Erwinia amylovora* [81], [82]. Conversely, mutations in T3E genes of the exchangeable effector locus (EEL) of *Pseudomonas syringae* are not associated to strong impairment of pathogenicity [79].

Our study contributes also to a better understanding of the evolutionary history of T3E genes within the *X. axonopodis* species. The core T3E genes set might represent the ancient T3E gene suite, acquired by the ancestor of the *X. axonopodis* species before diversification of pathovars, and thus before host specialization occurred. These core T3E genes might have evolved from this ancestor by vertical descent among *X. axonopodis* strains. However, some of these core T3E genes might have been acquired later in the evolution and then have been stably inherited along with the core genome. One can also postulate that among the core T3E genes set, some genes might have been lost during evolution in phylogenetically closely related pathovars, such as *xopE2* and *avrXacE3* in pathovars *manihotis* and *vasculorum* or as *xopQ* in pathovars *allii* and *ricini* (Figures 1 and 2). In contrast, the flexible T3E genes set might have evolved by horizontal gene transfer even though we cannot completely rule out gene loss during evolution. Analyses of *Xanthomonas* genomes clearly showed that these bacteria have been subjected to numerous horizontal gene transfers during evolution, sometimes from phylogenetically distant organisms [83], [84]. Moreover, gene acquisition is considered to be a major factor contributing to the genomic diversity of these bacteria but it seems that, once acquired, these genes are rarely transferred among lineages [85], [86]. Horizontal gene transfer events were supported by the fact that the majority of the variable T3E genes in our study cluster within pathogenicity islands in their *Xanthomonas* host genomes [8], [64], [87]. Indeed, these variable T3E genes exhibit a G+C content lower compared to the average value of the rest of the host bacterial genome, they are often associated with integrase genes, transfer RNA genes and/or IS elements or remnants of them, and they are found sometimes on plasmids. Regarding ubiquitous T3E genes, no linkage to pathogenicity islands can be detected since their G+C content is similar to the rest of their host bacterial genome, they are flanked by orthologous sequences, they are not associated with mobile elements, integrase or transfer RNA genes, and they reside on chromosome (except for *pthA1*).

Finally, the importance of knowing which T3E is ubiquitous or variable may be illustrated by the durability in the field of resistances introduced in crops. The pepper resistance gene *Bs2*, that matches the ubiquitous T3E *avrBs2* has been widely deployed in the field and still provides good level of resistance. On the other hand, prediction was made for low durability of the resistance conferred by *Bs1* that matches the variable T3E *avrBs1* [88].

### A correspondence between composition of T3E repertoires and pathovars of *X. axonopodis* supports a “repertoire for repertoire” hypothesis

The phylogeny of the strains we used in this study was constructed based on the sequence of *rpoD* housekeeping gene. Our results confirm that host specificity is not necessarily correlated to phylogeny [24], [35], [37]. Indeed, some pathovars are clearly polyphyletic, e. g. pathovars *phaseoli*, *dieffenbachiae*, *glycines* or *vasculorum*. However, the dendrogram constructed based on the T3E presence/absence matrix groups strains by pathovar (except for the pathovar *aurantifolii*), irrespective of the phylogenetic relationships between strains. For example, in the *rpoD* phylogeny, the pathovar *phaseoli* is scattered over the tree. In particular, the genetic lineage 1 highly diverges from the other lineages, as previously mentioned [24]. In contrast, on the dendrogram constructed on the matrix of presence/absence of T3Es, the four distinct genetic lineages identified in the pathovar *phaseoli* clustered together. Thus in our study, strains displaying a similar T3E repertoire belong to the same pathovar, even though they may be phylogenetically distant.

Conversely, strains displaying different host specialisation exhibit different T3E repertoires, even though these strains may be very close phylogenetically. For example, based on our *rpoD* phylogeny, strains belonging to the pathovar *vignicola* are mixed with strains belonging to the genetic lineage 2 of the pathovar *phaseoli*. However, their T3E repertoires are highly divergent, and strains do not display the same host range. Even more striking is the example of strains CFBP3541 and CFBP3835 that belong to the pathovars *citrumelo* and *alfalfae*, respectively. Phylogenetically, these strains are much closer to strains belonging to the pathovar *anacardii* or to the pathovar *phaseoli* than other strains of their respective pathovars. However, the T3E repertoire of strains CFBP3541 and CFBP3835 is identical or highly similar to that of other strains of pathovars *citrumelo* and *alfalfae*, respectively.

Such results support the hypothesis that T3E repertoires may explain a pathological convergence of phylogenetically distant strains. Thus, for a given strain, the T3E repertoire in its entirety would greatly determine the host range. Such hypothesis was also suggested by recent data obtained on a wide collection of strains of *Pseudomonas syringae* isolated from different host plants [16]. In addition, we performed an analysis of T3E gene history using parsimony as implemented in the Mesquite software package [89]. Parsimony method is particularly well suited for such binary data like presence or absence of T3E gene. Figure 1 shows that the trait “presence of the T3E gene *xac3090*” appears at several nodes in the phylogenetic tree. For example, it is shown that the occurrence of *xac3090* in the pathovar *glycines* probably results from multiple independent evolutionary events compatible with the hypothesis of an adaptive convergence for pathogenicity.

The variability observed in T3E repertoires between strains belonging to the same pathovar may explain race/cultivar specificity. Furthermore, in polyphyletic pathovars such as pathovar *phaseoli* the differences in repertoires observed between the four genetic lineages [24] of this pathovar may reflect differences in host range that was not revealed yet. One could think that pathovar *phaseoli* strains may have evolved diverse T3E repertoires to extend their host ranges or increase their survival on various unrelated plant species, as it was postulated for *Pseudomonas syringae* strains [90]. We now plan to thoroughly test host ranges of each genetic lineage of the pathovar *phaseoli* on plants belonging to the *Fabaceae* family in order to test such hypothesis.

Thus, our results support a “repertoire for repertoire” hypothesis as the molecular basis of host specificity of plant pathogenic bacteria. In such hypothesis, the outcome of the interaction between the bacterial pathogen and the plant would greatly depend on the confrontation of the repertoires of bacterial pathogenic determinants, such as T3E genes, and plant “guard” genes. Such hypothesis is compatible with the model proposed by Jones and Dangl [21], as well as with the fact that non-host resistance is constituted of multilayered basal defences that bacteria must overcome to induce disease [91], [92].

Our next goal will be to determine by Southern-blot hybridization whether T3E genes are present in multiple copies in our strain collection. Indeed, in *Pseudomonas syringae* pv. *tomato* strain DC3000, two copies of the *hopAM1* gene has been found [18], [93]. For T3E genes belonging to the *avrBs3*/*pthA* gene family, it is common to find more than 10 copies of these genes in strains of *Xanthomonas* such as *X. axonopodis* pv. *malvacearum*, *X. oryzae* pv. *oryzae* or *X. oryzae* pv. *oryzicola* [75]. The presence of such multiple copies of T3E genes within T3E repertoires may impact the host range of the strains. It has been reported that the contribution to pathogenicity in a given strain is not equal between the different *avrBs3*/*pthA* gene members: only a few members encode major virulence determinants whereas other members are potential reservoir genes providing sources for rapid evolution and adaptation in the event of host recognition [75].

However, one should keep in mind that, although T3E repertoires of plant pathogenic bacteria probably greatly impact their host range, other molecular determinants are also likely involved in host specificity and tissue specificity as well. In particular, early interactions such as host perception may also greatly impact host range in natural conditions. The importance of phenomena such as chemotaxis in the interactions between plant associated bacteria and their hosts has been widely documented. In the case of the plant pathogen *Ralstonia solanacearum*, for example, a chemotactic mutant is not able to colonize its host when inoculated in the soil, whereas it retains full pathogenicity when infiltrated directly in the plant tissues [94]. Hemagglutinin-related proteins, that appeared variable among *Ralstonia solanacearum* strains, are molecular determinants that could account for host specificity [12]. Furthermore, a recent comparative analysis of eight *Xanthomonas* genomes revealed that host- and tissue-specificity may result from subtle changes in a small number of individual genes in the *gum*, *hrp*, *xps*, *xcs* or *rpf* clusters and differences among regulatory targets, secretory substrates or genes for environmental sensing [38]. By analyzing amino acid residues, *hpaA* and *xpsD* have been revealed as candidate determinants of tissue specificity in *Xanthomonas* [38]. Since our study did not reveal correlation between T3E genes and tissue specificity, further sequencing of T3E genes and analysis of the T3E gene products polymorphisms are now required to identify new candidate determinants of tissue specificity.

Our results, which show a correspondence between composition of T3E repertoires and pathovars of *Xanthomonas*, do support the hypothesis that T3Es can affect host range in *Xanthomonas*. Nevertheless, our approach based on PCR and dot-blot hybridization methods is not sufficient to unequivocally consider that repertoires of T3Es determine host specificity in *Xanthomonas axonopodis* pathovars. To support the “repertoire for repertoire” hypothesis, we now plan to point our work towards functional studies based on our results.

### Typing the T3E repertoires of plant pathogenic bacteria may provide clues for functional studies on host specificity and insight into understanding the redundancy between T3Es

Repertoire of T3Es represents candidate determinants of host specificity of plant pathogenic bacteria since it has been shown that many T3Es can act as molecular double agents that betray the pathogen to plant defences in some interactions and suppress host defences in others [11], [20], [95]. T3Es have been shown to be involved in varietal resistance as well as in non-host resistance and they are reported to suppress both PTI (PAMP-triggered immunity) and ETI (effector-triggered immunity), the multilayered plant defences that bacteria must overcome to induce disease [21], [31], [91], [92], [96]–[98]. Thus, within a T3E repertoire, there are evidences of interplay among T3Es since they can suppress ETI [96], [99] and they can make redundant contributions to virulence [25], [26]. Moreover, individual T3E may contribute differently to the outcome of the infection on different hosts [90]. Comparisons of T3E repertoires in *Pseudomonas* strains lead to the conclusion that either different combinations of sequence-unrelated T3Es (or T3E alleles) with redundant functions or few common T3Es may promote successful pathogenesis by distinct strains on the same hosts [90], [93].

Our work provides clues for functional studies that will aim at showing gain or loss of function. For instance, focusing on strains of pathovars *vasculorum* and *manihotis* may be an excellent approach since strains of both pathovars have similar T3E repertoires (Figure 2) and the number of variable T3Es is not too important to reasonably set up functional studies for further analysis of the role of T3E repertoires in host specificity. It would be interesting to observe whether the deletion of the variable T3E gene, *xopB*, in pv. *vasculorum* or the transfer of this T3E gene to pv. *manihotis* narrow or enlarge the host range of the strains. The same kind of functional studies might be performed with the three variable T3E gene, *avrXv3*, *avrXccA2* and *avrRxo1* in pv. *manihotis*. Another example could be with strains CFBP1519 (pv. *glycines*) and CFBP3530 (pv. *aurantifolii*). Indeed, these strains are phylogenetically closely related since they exhibit the same *rpoD* sequence (Figure 1) and they harbour highly similar T3E repertoires since only two T3E genes discriminate both pathovars: *xopC* (present in pv. *glycines* and absent in pv. *aurantifolii*) and *avrXccB* (present in pv. *aurantifolii* and absent in pv. *glycines*) (Figure 2).

A major pitfall in deciphering the role of T3Es in the pathogenicity of plant pathogenic bacteria is that inactivation of a single T3E has often no detectable effect on pathogenicity. Functional redundancy among T3Es has largely been hypothesized to explain such phenomenon [25], [26]. Data provided in this study may help to better select strains for mutating single T3Es and combined T3Es to provide insight into the functional redundancy of T3Es that may have a role in the delineation of the host range of the strains. Several T3E families have been found within *Xanthomonas* genomes, such as the YopJ/AvrRxv family, the AvrBs3/PthA family, the HopX/AvrPphE family, the HopAE1 family or the PopC family [8], [11], [64], [68]. For instance, to reveal functional overlap between T3Es, one could focus on T3Es that belong to the YopJ/AvrRxv family of cysteine proteases since, in our study, we selected several T3Es (XopJ, AvrRxv, AvrBsT, XccB and AvrXv4) of this family [67], [68]. Noel and colleagues reported that a mutation of the T3E XopJ in *X. axonopodis* pv. *vesicatoria* strain 85-10 cannot be associated with any decrease in pathogenicity [78]. Genome sequence analysis [64] and results obtained in our study reveal that this strain carries AvrRxv, another cysteine protease of the same family that may partially complement an inactivation of XopJ. Furthermore, we show in the present study that both *xopJ* and *avrRxv* are frequently associated in the species *X. axonopodis* (Figure 4) even though they are not genetically linked [64]. It would now be interesting to construct a double mutant by deleting both *xopJ* and *avrRxv* in a *Xanthomonas axonopodis* strain in order to provide insight into the functional redundancy of these T3Es. Similarly, we constructed an *avrBsT* mutant in strain CFBP4834 of *X. axonopodis* pv. *phaseoli*. No phenotype could be associated to the mutation [our unpublished data]. But besides AvrBsT, the repertoire of the strain CFBP4834 also contains XccB, another cysteine protease of the YopJ/AvrRxv family. We also show that there is a high frequency of association between AvrBsT and XccB in the species *X. axonopodis* (Figure 4). However, some strains of the pathovar *mangiferaeindicae* or of the pathovar *alfalfae*, only display one cysteine protease of the YopJ/AvrRxv family. Selecting one of these strains may ease functional studies on T3Es of the YopJ/AvrRxv cysteine proteases in plant pathogenic bacteria. In order to carry out functional comparisons of T3Es, one could also use gene ontology annotations that do not only depend on sequence similarities [100]–[101]. Such an approach may highlight shared and divergent pathogenic strategies of T3Es deployed by the various pathovars of *X. axonopodis*. Our results will then help in the determination of redundant-effector groups (REGs) in *Xanthomonas* strains as it has been done recently in the *Pseudomonas syringae* pv. *tomato* strain DC3000 [26]. These authors clearly demonstrated that plant pathogenic bacteria have evolved the capacity to deliver into plant cells T3Es with very little sequence similarity that are redundant in function [26]. Another demonstration of sequence-unrelated T3Es that function in the same plant defense pathway is AvrRpm1, AvrRpt2 and AvrB, that are not recognized by the same resistance genes but all target the *Arabidopsis* RIN4 protein [11]. Since these three T3Es only rarely cooccur in *Pseudomonas syringae* strains, this suggests that convergent evolution is driven by the need to manipulate particular host proteins [11]. Finally, elucidation of functional overlaps between T3Es should help us understand how the diverse T3Es in a repertoire may function as a system in plant hosts and may shape the host range of the strains.

### Pathoadaptation of *X. axonopodis* strains is suggested by sequence variations revealed in some T3E genes

In regard to their central role in pathogenicity, T3Es are likely under strong selection pressures imposed by the defence system of the host plant. To escape plant defences, a pathogen may acquire new T3Es by horizontal gene transfer that would suppress defence reactions induced after recognition of the pathogen by the plant [99]. Alternatively, pathoadaptation of bacterial strains may occur through diverse mechanisms (single nucleotide polymorphism, insertion, deletion, or loss of a given T3E), to avoid being recognized by the host plant [102]–[106]. In the course of this study, we found DNA rearrangements that suggest pathoadaptation for *X. axonopodis* strains.

Our distribution study performed by the PCR amplification method allowed us to identify 23 DNA rearrangements within T3E genes. Interestingly, these DNA rearrangements were found only in T3E genes belonging to the accessory genome. If we consider that these variable genes may influence host specificity, such identified DNA rearrangements in some T3E genes might have a significant role in pathological adaptation of these plant pathogenic bacteria to their hosts. Among DNA rearrangements identified in the course of this study, there are a deletion within *xopF2* of one pathovar *aurantifolii* strain and a perfect tandem duplication within *xopD* of one pathovar *vesicatoria* strain. Interestingly, both DNA rearrangements do not shift the reading frames suggesting that these strains used these strategies to generate modified form of the XopF2 and XopD proteins to avoid recognition by the plant. Regarding *xopD*, to our knowledge, this is the first example of a T3E gene, except for genes belonging to the *avrBs3*/*pthA* gene family [75], exhibiting perfect tandem duplication within its nucleotide sequence. It is tempting to speculate that the tandem duplication in the *xopD* gene may affect the host adaptation of this pathovar *vesicatoria* strain. Indeed, in *Xanthomonas*, it has been reported that insertions or deletions in the central part, where tandem duplications reside, of T3Es belonging to the AvrBs3/PthA family induce alterations of the host range of the strains [75], [107], [108].

Another example of pathoadaptive evolution comes from the action of transposable elements. Indeed, we identified in the frame of this study several T3E genes that are disrupted by different IS elements. Numerous ISs have previously been found inserted into T3E genes among plant pathogenic bacteria, and some of them were shown to shift plant-pathogen interactions from incompatible to compatible [93], [105], [109]–[112]. In our study, the majority of identified IS elements belongs to the IS*3* family-IS*407* group whereas the remaining ones belong to the IS*5* and IS*1595* families. Interestingly, when looking at the flanking sequences of T3E genes in *Xanthomonas* sequenced genomes [8], [64], we only found IS elements that belong to the same three families, with again a large majority of ISs classified within the IS*3* family - IS*407* group. Furthermore, another IS element (IS*476*) belonging to the IS*3* family-IS*407* group has been disclosed in the *avrBs1* gene within one *X. axonopodis* pv. *vesicatoria* strain [113]. Altogether, these observations suggest that, in *Xanthomonas* strains, members of these three IS families might play an important role in T3E gene evolution since these mobile elements may alter their expression, they may be involved in their mobility as well as in the terminal reassortment process [71], [109], [114]–[118]. Otherwise, it is also striking to note that some IS elements belonging to the IS*3* family-IS*407* group have been found just downstream PIP boxes, the binding motif for the transcriptional regulator HrpX [119], in the sequenced genome of *X. axonopodis* pv. *vesicatoria* [64]. It is thus tempting to speculate that the transposition of such replicative IS elements [115], and then the subsequent inactivation of a given T3E gene, might be co-regulated with the *hrp* genes cluster. Thus, one can reasonably think that inactivation of some T3E genes by IS elements might be of importance in host adaptation for plant pathogenic bacteria. To verify this hypothesis, it would be interesting to focus for instance on *avrXv3* since it is altered by IS*1595* in all tested pathovar *alfalfae* strains. We plan to complement these strains with a functional *avrXv3* gene in order to observe a modification of the interaction between the pathovar *alfalfae* strains and their hosts. It would also be interesting to focus on *xopC* in pathovar *mangiferaeindicae* strains since this gene is altered in strains isolated from *Schinus terebenthifolius* but not in those isolated from *Mangifera indica* (Figure 5). Since strains from both hosts of isolation exhibit identical T3E gene repertoires, the functional complementation of *xopC* might lead to a modification in host adaptation of these strains.

The finding that multiple T3E genes are affected by DNA rearrangements raises the question of the functionality of these genes within the repertoires. Our approach allowed us to show that several T3E genes are likely inactive since they are disrupted by ISs or exhibit a frameshift mutation leading to a premature stop codon. But, T3E genes may be non-functional for other reasons that we did not challenge by our approach, such as lack of expression or inability to translocate T3E proteins. Schechter and colleagues, in 2006, by using multiple approaches on the T3E repertoire of the *Pseudomonas syringae* pv. *tomato* strain DC3000, revealed that 33 T3Es are likely to be active, 12 T3Es are likely to be inactive and 8 T3Es may or may not be produced at functional levels [18]. It will be now important to check the functionality of each T3E gene in the repertoires that may impact the host range of *Xanthomonas* strains. Knowing whether a T3E gene is active or inactive is of interest for evolutionary studies. Indeed, it is possible that selection pressure for the inactivation of a T3E gene may result from the acquisition of new gene functions in both the host and the bacterium and that loss of function may be an important factor in the evolution of *Xanthomonas axonopodis* virulence. Loss of gene function may be beneficial to bacterial strains and it is considered to be a contributing factor to the evolution of virulence of many pathogens [104], [105], [120].

Finally, our results do support the hypothesis that T3E repertoires can affect the host range of *Xanthomonas* strains and that the evolution of T3E repertoires is driven by the need for interactions among T3Es as they co-ordinately disarm multiple layers of plant defenses. Our results also support the hypothesis that the evolution of T3E repertoires is also likely driven by the exposure to diverse resistance mechanisms in plants. So, the evolution and function of T3Es in a repertoire may be influenced by a co-evolutionary arms race between pathogens and hosts [105]. The second hypothesis is supported by the observation that a T3E loss and then the evolution of a T3E repertoire can be driven by exposure to host defence system [121]. Recently, it has been proposed that the host defence can accelerate the generation of genomic rearrangements that provide selecting advantage to the pathogen [104]. Therefore, it is tempting to speculate that the numerous DNA rearrangements found in T3E genes from *Xanthomonas* strains in the course of our study may be the result of exposure to various host plants. In that case, one could speculate that pathogens in response to selection pressure imposed by host defence systems, may have driven the inactivation of some T3E genes by insertion of ISs, or the modification of other T3Es by in-frame deletion or perfect tandem duplication. These DNA rearrangements may have had a significant role in avoidance of host recognition and then in shaping the host range of the *Xanthomonas* strains. It is also reported that similar exposure of bacterial strains to environmental stress outside the host could also drive the horizontal transfer of T3Es from ecologically related plant pathogens that could lead to evolution of T3E repertoires and then of bacterial pathogenicity towards plants [104], [105].

### Perspectives

Our results provide resources for functional studies on host specificity of plant pathogenic bacteria. Our work will help to select strains to study the role of single or combination of T3E genes in the interaction with plants, as well as for studies aiming at understanding the molecular mechanisms of redundancy between T3Es. Moreover, the discovery of genetic rearrangements in genes encoding T3Es demonstrates the importance of looking at the allelic diversity of T3Es as well as at the expression of these genes. Indeed, impact of genetic rearrangements in T3E genes on host range has recently been well documented [105], [106]. Thus, we plan to continue to analyze the allelic diversity of T3Es in our collection of *Xanthomonas* strains for evolutionary studies. Furthermore, our results strongly suggest that determination of T3E repertoires may be used for identification of *Xanthomonas* strains at the pathovar level. Thus, we will aim at developing a diagnostic tool for such purpose.

Finally, our study illustrates the importance of distribution analyses of virulence-associated genes by using large collections of bacterial strains. This approach can be useful for the identification of the candidate determinants of host specificity. For instance, it could be useful to perform such investigation on large collections of *E. coli* strains that are pathogenic on poultry or on humans. Indeed, no set of virulence genes was clearly identified yet to discriminate between avian and human strains [3]. To our knowledge, if presence or absence of genes of the T3SS was analyzed, repertoires of T3E were not yet compared. But interestingly, similarly to what was found for plant pathogenic bacteria, T3E genes appeared as differential genes in SSH between avian and human strains [2].

## Materials and Methods

### Bacterial strains

A collection of 132 strains of *X. axonopodis* [32] belonging to 18 different pathovars were included in this study (Table 1). The bacterial strains were obtained from international culture collections (mostly the French Collection of Plant pathogenic Bacteria; http://www.angers.inra.fr/cfbp/). Since, in the scope of this study, it was not possible to determine the host range of each strain, we decided to select only well characterized pathovars for which host range has been determined precisely and published data are available [40]–[56]. Among the 18 selected pathovars, 7 are considered as vascular pathogens and 11 are considered as non-vascular pathogens (http://www.cababstractsplus.org/) [40]–[56]. Furthermore, strains were selected carefully in order to get the largest diversity. Thus, for each pathovar, strains were chosen from various geographical origins, hosts and years of isolation. Each pathovar is represented by at least 5 strains, except for the pathovar *axonopodis* for which only two available strains, including the type strain of the species *X. axonopodis* (CFBP4924), could be found in international collections. More strains belonging to the pathovar *phaseoli*, the bacterial model of our team [24], [53]–[56], were selected since it is a genetically diverse and polyphyletic pathovar, four genetic lineages were disclosed that are not closely related [24]. In this study, the pathovar *phaseoli* was then used to highlight the potential convergence of T3E repertoires of phylogenetically unrelated strains. The 18 selected pathovars represent the overall diversity within the *X. axonopodis* species since they were selected from the 6 defined rep-PCR clusters (from 9.1 to 9.6) of this species (Figure 1) [34], [35]. Moreover, we also included *Escherichia coli* strain DH5α and *Xanthomonas* strains whose genome has been sequenced (*X. axonopodis* pv. *vesicatoria* strain CFBP5618; *X. axonopodis* pv. *citri* strain 306, *X. campestris* pv. *campestris* strains CFBP5241 and CFBP6650, *X. oryzae* pv. *oryzae* strain CFBP7088, *X. oryzae* pv. *oryzicola* strain CFBP7109 and *X. albilineans* strain CFBP7063) (http://www.genomesonline.org/) [8], [64]–[66]. We also included in our study *X. axonopodis* pv. *phaseoli* strain CFBP4834 whose genome has been sequenced recently (our unpublished data). All of these sequenced strains were used as positive or negative controls for PCRs and dot-blot hybridizations.

### DNA extraction

Genomic DNA was extracted from all bacterial strains grown overnight at 28°C in YP medium (yeast extract, 7 g/liter; peptone, 7 g/liter) by using the standard hexadecyltrimethylammonium bromide method [122]. Quality and quantity of DNA was spectrophotometrically evaluated (Nanodrop ND-1000, Nanodrop Technologies).

### Selection of T3E genes

Table S2 presents the complete list of the 35 T3E genes included in this study. Selected genes comprised those identified from the sequenced genomes of *Xanthomonas* strains (17 from *X. axonopodis* pv. *vesicatoria* strain CFBP5618, 8 from *X. campestris* pv. *campestris* strain CFBP5241 and 7 from *X. axonopodis* pv. *citri* strain 306) [8], [64]. We also selected 3 avirulence genes from other *X. axonopodis* pv. *vesicatoria* strains whose genome has not been sequenced: *avrBsT* from strain 75-3 [123] and *avrXv3* and *avrXv4* from strain 91-118 [124], [125]. Some of the selected T3E genes are members of the defined T3E families in bacterial pathogens such as the AvrRxv/YopJ (C55) family of cysteine proteases, the AvrBs3/PthA family of transcriptional activators, the PopC family of Leucin-Rich Repeats proteins, the HopAE1 family and the HopX/AvrPphE family (Table S2) [8], [11], [64], [68]. The other selected T3E genes have unknown functions to date. It is important to note that in the present study we tried to be as exhaustive as possible since we selected T3E genes not from only one but from 5 different *Xanthomonas* strains that belong to diverse species and pathovars. We followed this approach to minimize unavoidable bias of this kind of analysis; indeed it is certain that unidentified T3E genes may reside in *Xanthomonas* strains whose genome has not been sequenced yet.

### PCR amplifications

Two complementary approaches were undertaken to characterize the T3E repertoires of our collection of strains: PCR and dot blot hybridization. The presence or the absence of an ortholog of each selected T3E gene was first determined by PCR. All *X. axonopodis* strains were first submitted to a PCR analysis by using specific T3Es primers. Pairs of primers (Table S2) were designed from the DNA sequences of the selected T3E genes available in databases. All of these primers pairs allowed the amplification of the full-length T3E DNA sequence, except for the *avrBs2* and *avrBs3* genes for which only partial DNA sequences were amplified (Table S2). The *Xanthomonas* strains, from which T3E genes were selected, were taken as positive or negative controls for all PCR experiments. PCR amplifications were carried out with a 20 µl reaction mixture containing 1× Go Taq Buffer (Promega), 200 µM dNTP, 0.5 µM of each primer, 0.4 U of Go Taq Polymerase (final concentrations) and 1 ng of template genomic DNA. The amplification conditions using the T3E primers were 2 min of initial denaturation at 94°C; followed by 30 cycles of 94°C for 1 min, 60°C for 1 minute and 72°C for 2 min; with a final extension of 10 min at 72°C. A reaction was considered as positive if a single clear band with the expected size was detected onto agarose gels. When a single band with an unexpected size was observed, the amplified PCR product was recovered from the gel and then sequenced (see below).

### Dot blot hybridizations

As sequence variation may occur between T3E orthologs, thus preventing annealing of the PCR primers used, presence or absence of an ortholog was then confirmed by nucleic acid hybridization. For each dot blot hybridization experiment, we included, as negative and positive controls, water, *E. coli* strain DH5α and *Xanthomonas* strains whose genome has been sequenced (see above for strain details).

Hybridization probes were obtained by PCR amplification of the selected T3E genes from the sequenced genome of *Xanthomonas* strains using specific primers listed in Table S2. Probes were labelled using the PCR Digoxigenin (DIG) labelling mix (Roche Applied Science, France). PCR reactions contained 200 µM dNTP-DIG, and the other components as above. PCR for preparation of DIG-labeled DNA probes was performed in a themocycler programmed for denaturation at 94°C for 2 min and then for 30 cycles of 94°C for 1 min, 60°C for 1 min, 72°C for 2 min and finally 72°C for 10 min. The PCR products were purified by using the Nucleospin® extract II kit (Macherey- Nagel Hoerdt, France).

Genomic DNA (250 ng) of each strain was transferred to Biodyne® N+ membranes (Pall Gelman Laboratory). DNAs were randomised onto membranes. Prehybridization, hybridization and detection were carried out by using the DIG Labelling and Detection Starter kit (Roche Applied Science, France) according to the manufacturer's instructions. Hybridizations were performed overnight at 42°C. To ensure high stringency, membranes were washed twice for 15 min in 2**×** SSC and 0.1% SDS buffer and twice in 0.1**×** SSC and 0.1% SDS buffer at 68°C. Hybridization signals were detected using the Fab fragments of an anti 1-2 digoxigenin antibody conjugated with alkaline phosphatase (Anti-DIG-AP) and Nitro-Blue Tetrazolium Chloride/5-Bromo-4-Chloro-3′-Indolyphosphate p-Toluidine Salt (NBT/BCIP). A subset of hybridization experiments was replicated twice to assess the reproducibility of the dot blot results. Furthermore, we assessed the robustness of our approach by using sequenced *Xanthomonas* strains. Indeed, for these strains we were able to compare the T3E repertoires obtained by PCR and dot-blot hybridization with the expected T3E repertoires based on the genome sequence. For each of these strains, the obtained T3E repertoire corresponded to the expected T3E repertoire. This approach combined with BLAST analyses (http://blast.ncbi.nlm.nih.gov/Blast.cgi) allowed us to determine that the minimum identity at the DNA level between a T3E on the membrane and a T3E ortholog in the probe had to be at least 71% to give a signal above background.

### DNA Sequencing and sequence analysis of amplified T3E genes from *X. axonopodis* strains

The amplified PCR products were purified from agarose gels with the Nucleospin® extract II kit (Macherey-Nagel EURL, France) and then sequenced at Genoscreen genomic analysis platform (Lille, France). Sequence data were examined by using the BLAST search programs (http://blast.ncbi.nlm.nih.gov/Blast.cgi), the Mobyle portal (http://mobyle.pasteur.fr/cgi-bin/MobylePortal/portal.py) and the IS Finder database (http://www-is.biotoul.fr/is.html).

### *rpoD* amplification, sequencing and sequence analysis

The phylogenetic analysis of *X. axonopodis* strains was performed by sequencing the housekeeping *rpoD* gene (RNA polymerase sigma-70 factor). Primers were designed from the *rpoD* sequence of the *X. campestris* pv. *campestris* strain CFBP 5241 (GenBank accession no. NP639081) (Table S2). PCR amplifications were performed in a total volume of 50 µl using 3 ng of genomic DNA, 200 µM dNTPs, 0.5 µM of each primer, 1.5 mM MgCl_2_ and 0.4 U of Go *Taq* polymerase in 1× Colorless Go Taq buffer (Promega). The PCR cycling conditions consisted of an initial denaturation step at 94°C for 5 min, followed by 30 cycles of 94°C for 30 s, 60°C for 60 s, 72°C for 30 s, with a final extension step at 72°C for 7 min. PCR amplicons were then sent to Ouest Genopole sequencing platform (Nantes, France). Forward and reverse sequences were obtained by using the *rpoD* specific PCR primers. These sequences were edited and assembled by using PREGAP 4 and GAP 4 of the Staden Package [126]. All *rpoD* sequences were then aligned using BioEdit (http://www.mbio.ncsu.edu/BioEdit/bioedit.html). All *rpoD* sequences have been deposited in the GenBank data library (accession numbers from FJ561596 to FJ561725).

### Data analysis

Based on the presence/absence matrix of T3E genes for each of the 132 strains of *X. axonopodis*, we constructed a dendrogram using Jaccard distances and Neighbor-Joining method. Bootstrapping was performed with 1,000 replicates to assess the robustness of our dendrogram. The resulting dendrogram was visualised using the PAST 1.81 software (http://folk.uio.no/ohammer/past/).

Phylogenetic trees based on the *rpoD* sequences analysis were constructed by using maximum likelihood method. Best nucleotide substitution model was found using Modeltest v.3.7 [62]. Akaike Information Criterion (AIC) was used for model selection. Parameters of the selected model were used for maximum likelihood heuristic search using PAUP*4.0 beta10 [127]. Confidence on node was assessed by bootstrapping 1000 times.

Based on the presence/absence matrix of T3E genes, we calculated frequencies of association between T3E genes within *X. axonopodis* strains. We retained only cases where both T3E genes are present in the same strains. Furthermore, to analyze T3E gene histories, we used the parsimony method as implemented in the Mesquite software package [89].

## Supporting Information

Table S1 Percentage of presence of each T3E gene in the various pathovars of X. axonopodis.
(0.03 MB XLS)

Table S2 List of genes analyzed in this study: gene functions and primers sequences used for PCR amplifications and for making probes for dot-blot hybridizations.
(0.03 MB XLS)

## References

[1] Moulin-Schouleur M, Répérant M, Laurent S, Brée A, Mignon-Grasteau S (2007). Extraintestinal pathogenic *Escherichia coli* strains of avian and human origin: link between phylogenetic relationships and common virulence patterns.. J Clin Microbiol.

[2] Kariyawasam S, Scaccianoce JA, Nolan LK (2007). Common and specific genomic sequences of avian and human extraintestinal pathogenic *Escherichia coli* as determined by genomic subtractive hybridization.. BMC Microbiol.

[3] Ron EZ (2006). Host specificity of septicemic *Escherichia coli*: human and avian pathogens.. Curr Opin Microbiol.

[4] Mokady D, Gophna U, Ron EZ (2005). Virulence factors of septicemic *Escherichia coli* strains.. Int J Med Microbiol.

[5] Mokady D, Gophna U, Ron EZ (2005). Extensive gene diversity in septicemic *Escherichia coli* strains.. J Clin Microbiol.

[6] Dye DW, Bradbury JF, Goto M, Hayward AC, Lelliot RA (1980). International standards for naming pathovars of phytopathogenic bacteria and a list of pathovar names and pathotype strains.. Rev Plant Pathol.

[7] Flor HH (1956). The complementary genetic systems in flax and flax rust.. Adv Genet.

[8] da Silva ACR, Ferro JA, Reinach FC, Farah CS, Furlan LR (2002). Comparison of the genomes of two *Xanthomonas* pathogens with differing host specificities.. Nature.

[9] Ochiai H, Inoue Y, Takeya M, Sasaki A, Kaku H (2005). Genome sequence of *Xanthomonas oryzae* pv. oryzae suggests contribution of large numbers of effector genes and insertion sequences to its race diversity.. JARQ.

[10] Feil H, Feil WS, Chain P, Larimer F, DiBartolo G (2005). Comparison of the complete genome sequences of *Pseudomonas syringae* pv. *syringae* B728a and pv. *tomato* DC3000.. Proc Natl Acad Sci U S A.

[11] Grant SR, Fisher EJ, Chang JH, Mole BM, Dangl JL (2006). Subterfuge and manipulation: Type III effector proteins of phytopathogenic bacteria.. Annu Rev Microbiol.

[12] Guidot A, Prior P, Schoenfeld J, Carrère S, Genin S (2007). Genomic structure and phylogeny of the plant pathogen *Ralstonia solanacearum* inferred from gene distribution analysis.. J Bacteriol.

[13] He YQ, Zhang L, Jiang BL, Zhang ZC, Xu RQ (2007). Comparative and functional genomics reveals genetic diversity and determinants of host specificity among reference strains and a large collection of Chinese isolates of the phytopathogen *Xanthomonas campestris* pv. *campestris*.. Genome Biol.

[14] Demuth A, Aharonowitz Y, Bachmann TT, Blum-Oehler G, Buchrieser C (2008). Pathogenomics: an updated European Research Agenda.. Infect Genet Evol.

[15] Hayward A, Swings J, Civerolo EL (1993). The hosts of *Xanthomonas*.. *Xanthomonas*.

[16] Sarkar SF, Gordon JS, Martin GB, Guttman DS (2006). Comparative genomics of host-specific virulence in *Pseudomonas syringae*.. Genetics.

[17] Chang JH, Urbach JM, Law TF, Arnold LW, Hu A (2005). A highthroughput, near-saturating screen for type III effector genes from *Pseudomonas syringae*.. Proc Natl Acad Sci U S A.

[18] Schechter LM, Vencato M, Jordan KL, Schneider SE, Schneider DJ (2006). Multiple approaches to a complete inventory of *Pseudomonas syringae* pv. *tomato* DC3000 type III secretion system effector proteins.. Mol Plant-Microbe Interact.

[19] Gürlebeck D, Thieme F, Bonas U (2006). Type III effector proteins from the plant pathogen *Xanthomonas* and their role in the interaction with the host plant.. J Plant Physiol.

[20] da Cunha L, Sreerekha MV, Mackey D (2007). Defense suppression by virulence effectors of bacterial phytopathogens.. Curr Opin Plant Biol.

[21] Jones JD, Dangl JL (2006). The plant immune system.. Nature.

[22] Castaneda A, Reddy JD, El-Yacoubi B, Gabriel DW (2005). Mutagenesis of all eight *avr* genes in *Xanthomonas campestris* pv. *campestris* had no detected effect on pathogenicity but one *avr* gene affected race specificity.. Mol Plant Microbe Interact.

[23] Triplett LR, Zhao Y, Sundin GW (2006). Genetic differences between blight-causing *Erwinia* species with differing host specificities, identified by suppression subtractive hybridization.. Appl Environ Microbiol.

[24] Alavi SM, Sanjari S, Durand F, Brin C, Manceau C (2008). Assessment of the genetic diversity of *Xanthomonas axonopodis* pv. *phaseoli* and *Xanthomonas fuscans* subsp. *fuscans* as a basis to identify putative pathogenicity genes and a type III secretion system of the SPI-1 family by multiple suppression subtractive hybridizations.. Appl Environ Microbiol.

[25] Badel JL, Shimizu R, Oh H-S, Collmer A (2006). A *Pseudomonas syringae* pv. *tomato* avrE1/hopM1 mutant is severely reduced in growth and lesion formation in tomato.. Mol Plant Microbe Interact.

[26] Kvitko BH, Park DH, Velasquez AC, Wei C-F, Russell AB (2009). Deletions in the repertoire of *Pseudomonas syringae* pv. *tomato* DC3000 type III secretion effector genes reveal functional overlap among effectors.. PLoS Pathog.

[27] Badel JL, Nomura K, Bandyopadhyay S, Shimizu R, Collmer A (2003). *Pseudomonas syringae* pv. *tomato* DC3000 HopPtoM (CEL ORF3) is important for lesion formation but not growth in tomato and is secreted and translocated by the Hrp type III secretion system in a chaperone-dependent manner.. Mol Microbiol.

[28] Lopez-Solanilla E, Bronstein PA, Schneider AR, Collmer A (2004). HopPtoN is a *Pseudomonas syringae* Hrp (type III secretion system) cysteine protease effector that suppresses pathogen-induced necrosis associated with both compatible and incompatible plant interactions.. Mol Microbiol.

[29] Reboutier D, Frankart C, Briand J, Biligui B, Rona JP (2007). Antagonistic action of harpin proteins: HrpWea from *Erwinia amylovora* suppresses HrpNea-induced cell death in *Arabidopsis thaliana*.. J Cell Sci.

[30] Liao AP, Petrof EO, Kuppireddi S, Zhao Y, Xia Y (2008). *Salmonella* type III effector AvrA stabilizes cell tight junctions to inhibit inflammation in intestinal epithelial cells.. PLoS ONE.

[31] Zhao B, Ardales EY, Raymundo A, Bai J, Trick HN (2004). The *avrRxo1* gene from the rice pathogen *Xanthomonas oryzae* pv. *oryzicola* confers a nonhost defense reaction on maize with resistance gene *Rxo1.*. Mol Plant Microbe Interact.

[32] Vauterin L, Hoste B, Kersters K, Swings J (1995). Reclassification of *Xanthomonas*.. International Journal of Systematic Bacteriology.

[33] Vauterin L, Rademaker J, Swings J (2000). Synopsis on the taxonomy of the genus *Xanthomonas*.. Phytopathology.

[34] Rademaker JLW, Hoste B, Louws FJ, Kersters K, Swings J (2000). Comparison of AFLP and rep-PCR genomic fingerprinting with DNA-DNA homology studies: *Xanthomonas* as a model system.. Int J Syst Appl Microbiol.

[35] Rademaker JLW, Louws FJ, Schultz MH, Rossbach U, Vauterin L (2005). A comprehensive species to strain taxonomic framework for *Xanthomonas*.. Phytopathology.

[36] Fargier E (2007). L'étude de la pathologie de *Xanthomonas campestris* et de la structure génétique de ses pathovars a permis l'amélioration de la détection du pathogène dans les semences de Brassicacées..

[37] Young JM, Park DC, Shearman HM, Fargier E (2008). A multilocus sequence analysis of the genus *Xanthomonas*.. Syst Appl Microbiol.

[38] Lu H, Patil P, Van Sluys M-A, White FF, Ryan RP (2008). Acquisition and evolution of plant pathogenesis-associated gene clusters and candidate determinants of tissue-specificity in *Xanthomonas*.. PLoS ONE.

[39] Meyer DF, Bogdanove AJ, Jackson (2009). Genomics-driven advances in *Xanthomonas* biology.. Plant Pathogenic Bacteria: Genomics and Molecular Biology.

[40] Ah-You N, Gagnevin L, Chiroleu F, Jouen E, Rodrigues Neto J (2007). Pathological variations within *Xanthomonas campestris* pv. *mangiferaeindicae* support its separation into three distinct pathovars that can be distinguished by amplified fragment length polymorphism.. Phytopathology.

[41] Brunings AM, Gabriel DW (2003). *Xanthomonas citri*: breaking the surface.. Mol Plant Pathol.

[42] Roumagnac P, Gagnevin L, Gardan L, Sutra L, Manceau C (2004). Polyphasic characterization of xanthomonads isolated from onion, garlic and Welsh onion (*Allium* spp.) and their relatedness to different *Xanthomonas* species.. Int J Syst Evol Microbiol.

[43] Jones JB, Stall RE, Bouzar H (1998). Diversity among xanthomonads pathogenic on pepper and tomato.. Annu Rev Phytopathol.

[44] Berthier Y, Verdier V, Guesdon JL, Chevrier D, Denis JB (1993). Characterization of *Xanthomonas campestris* Pathovars by rRNA Gene Restriction Patterns.. Appl Environ Microbiol.

[45] Gonzalez C, Restrepo S, Tohme J, Verdier V (2002). Characterization of pathogenic and nonpathogenic strains of *Xa nthomonas axonopodis* pv. *manihotis* by PCR-based DNA fingerprinting techniques.. FEMS Microbiol Lett.

[46] Mkandawire ABC, Mabagala RB, Guzman P, Gepts P, Gilbertson RL (2004). Genetic diversity and pathogenic variation of common blight bacteria (*Xanthomonas campestris* pv. *phaseoli* and *X. campestris* pv. *phaseoli* var. *fuscans*) suggests pathogen coevolution with the common bean.. Phytopathology.

[47] Khatri-Chhetri GB, Wydra K, Rudolph K (2003). Metabolic diversity of *Xanthomonas axonopodis* pv. *vignicola*, casual agent of cowpea bacterial blight and pustule.. Eur J Plant Pathol.

[48] Restrepo S, Vélez CM, Verdier V (2000). Measuring the genetic diversity of *Xanthomonas axonopodis* pv. *manihotis* within different fields in Colombia.. Phytopathology.

[49] Gent DH, Al-Saadi A, Gabriel DW, Louws FJ, Ishimaru CA (2005). Pathogenic and genetic relatedness among *Xanthomonas axonopodis* pv. *allii* and other pathovars of *X. axonopodis*.. Phytopathology.

[50] Robène-Soustrade I, Laurent P, Gagnevin L, Jouen E, Pruvost O (2006). Specific detection of *Xanthomonas axonopodis* pv. *dieffenbachiae* in anthurium (*Anthurium andreanum*) tissues by nested PCR.. Appl Environ Microbiol.

[51] De Feyter R, Gabriel DW (1991). At least six avirulence genes are clustered on a 90-kilobase plasmid in *Xanthomonas campestris* pv. *malvacearum*.. Mol Plant-Microbe Interact.

[52] Zomorodian A, Rudolph K, Swings JG, Civerolo EL (1993). *Xanthomonas campestris* pv. *malvacearum*: cause of bacterial blight of cotton.. *Xanthomonas*.

[53] Jacques MA, Josi K, Darrasse A, Samson R (2005). *Xanthomonas axonopodis* pv. *phaseoli* var. *fuscans* is aggregated in stable biofilm population sizes in the phyllosphere of field-grown beans.. Appl Environ Microbiol.

[54] Alavi SM, Poussier S, Manceau C (2007). Characterization of IS*Xax1*, a novel insertion sequence restricted to *Xanthomonas axonopodis* pv. *phaseoli* (variants fuscans and non-fuscans) and *Xanthomonas axonopodis* pv. *vesicatoria*.. Appl Environ Microbiol.

[55] Darsonval A, Darrasse A, Meyer D, Demarty M, Durand K (2008). The type III secretion system of *Xanthomonas fuscans* subsp. *fuscans* is involved in the phyllosphere colonization process and in transmission to seeds of susceptible beans.. Appl Environ Microbiol.

[56] Darsonval A, Darrasse A, Durand K, Bureau C, Cesbron S (2009). Adhesion and fitness in the bean phyllosphere and transmission to seeds of *Xanthomonas fuscans* subsp. *fuscans.*. Mol Plant-Microbe Interact.

[57] Xu RQ, Blanvillain S, Feng JX, Jiang BL, Li XZ (2008). AvrAC(Xcc8004), a type III effector with a leucine-rich repeat domain from *Xanthomonas campestris* pathovar *campestris* confers avirulence in vascular tissues of *Arabidopsis thaliana* ecotype Col-0.. J Bacteriol.

[58] Maiden MCJ, Bygraves JA, Feil E, Morelli G, Russell JE (1998). Multilocus sequence typing: a portable approach to the identification of clones within populations of pathogenic microorganisms.. Proc Natl Acad Sci USA.

[59] Enright MC, Spratt BG (1999). Multilocus sequence typing.. Trends Microbiol.

[60] Urwin R, Maiden MCJ (2003). Multi-locus sequence typing: a tool for global epidemiology.. Trends Microbiol.

[61] Sarkar SF, Guttman DS (2004). Evolution of the core genome of *Pseudomonas syringae*, a highly clonal, endemic plant pathogen.. Appl Environ Microbiol.

[62] Posada D, Crandall KA (1998). Modeltest: testing the model of DNA substitution.. Bioinformatics.

[63] Cornelis GR (2006). The type III secretion injectisome.. Nat Rev Microbiol.

[64] Thieme F, Koebnik R, Bekel T, Berger C, Boch J (2005). Insights into genome plasticity and pathogenicity of the plant pathogenic bacterium *Xanthomonas campestris* pv. *vesicatoria* revealed by the complete genome sequence.. J Bacteriol.

[65] Lee BM, Park YJ, Park DS, Kang HW, Kim JG (2005). The genome sequence of *Xanthomonas oryzae* pathovar *oryzae* KACC10331, the bacterial blight pathogen of rice.. Nucleic Acids Res.

[66] Qian W, Jia Y, Ren SX, He YQ, Feng JX (2005). Comparative and functional genomic analyses of the pathogenicity of phytopathogen *Xanthomonas campestris* pv. *campestris*.. Genome Res.

[67] Hotson A, Mudgett MB (2004). Cysteine proteases in phytopathogenic bacteria: Identification of plant targets and activation of innate immunity.. Curr Opin Plant Biol.

[68] Kay S, Bonas U (2009). How *Xanthomonas* type III effectors manipulate the host plant.. Curr Opin Microbiol.

[69] Guttman DS, Vinatzer BA, Sarkar SF, Ranall MV, Kettler G (2002). A functional screen for the type III (Hrp) secretome of the plant pathogen *Pseudomonas syringae*.. Science.

[70] Rohmer L, Guttman DS, Dangl JL (2004). Diverse evolutionary mechanisms shape the type III effector virulence factor repertoire in the plant pathogen *Pseudomonas syringae*.. Genetics.

[71] Stavrinides J, McCann HC, Guttman DS (2008). Host-pathogen interplay and the evolution of bacterial effectors.. Cell Microbiol.

[72] Kearney B, Staskawicz BJ (1990). Widespread distribution and fitness contribution of *Xanthomonas campestris* avirulence gene *avrBs2*.. Nature.

[73] Duan YP, Castañeda A, Zhao G, Erdos G, Gabriel DW (1999). Expression of a single, host-specific, bacterial pathogenicity gene in plant cells elicits division, enlargement, and cell death.. Mol Plant Microbe Interact.

[74] Roden JA, Belt B, Ross JB, Tachibana T, Vargas J (2004). A genetic screen to isolate type III effectors translocated into pepper cells during *Xanthomonas* infection.. Proc Natl Acad Sci USA.

[75] Yang B, White FF (2004). Diverse members of the AvrBs3/PthA family of type III effectors are major virulence determinants in bacterial blight disease of rice.. Mol Plant Microbe Interact.

[76] Metz M, Dahlbeck D, Morales CQ, Al Sady B, Clark ET (2005). The conserved *Xanthomonas campestris* pv. *vesicatoria* effector protein XopX is a virulence factor and suppresses host defense in *Nicotiana benthamiana*.. Plant J.

[77] Jiang BL, He YQ, Cen WJ, Wei HY, Jiang GF (2008). The type III secretion effector XopXccN of *Xanthomonas campestris* pv. *campestris* is required for full virulence.. Res Microbiol.

[78] Noël L, Thieme F, Gäbler J, Büttner D, Bonas U (2003). XopC and XopJ, two novel type III effector proteins from *Xanthomonas campestris* pv. *vesicatoria*.. J Bacteriol.

[79] Alfano JR, Charkowski AO, Deng WL, Badel JL, Petnicki-Ocwieja T (2000). The *Pseudomonas syringae* Hrp pathogenicity island has a tripartite mosaic structure composed of a cluster of type III secretion genes bounded by exchangeable effector and conserved effector loci that contribute to parasitic fitness and pathogenicity in plants.. Proc Natl Acad Sci USA.

[80] Lorang JM, Shen H, Kobayashi D, Cooksey D, Keen NT (1994). *avrA* and *avrE* in *Pseudomonas syringae* pv. *tomato* PT23 play a role in virulence on tomato plants.. Mol Plant Microbe Interact.

[81] Gaudriault S, Malandrin L, Paulin JP, Barny MA (1997). DspA, an essential pathogenicity factor of *Erwinia amylovora* showing homology with AvrE of *Pseudomonas syringae*, is secreted via the Hrp secretion pathway in a DspB-dependent way.. Mol Microbiol.

[82] Bogdanove AJ, Bauer DW, Beer SV (1998). *Erwinia amylovora* secretes DspE, a pathogenicity factor and functional AvrE homolog, through the *hrp* (type III secretion) pathway.. J Bacteriol.

[83] Lima W, Van Sluys MA, Menck CF (2005). Non-gamma Proteobacteria gene islands contribute to the *Xanthomonas* genome.. Omics.

[84] Comas I, Moya A, Azad RK, Lawrence JG, Gonzalez-Candelas F (2006). The evolutionary origin of Xanthomonadales genomes and the nature of the horizontal gene transfer process.. Mol Biol Evol.

[85] Gillings MR, Holley MP, Stokes HW, Holmes AJ (2005). Integrons in *Xanthomonas*: A source of species genome diversity.. Proc Natl Acad Sci USA.

[86] Lerat E, Daubin V, Ochman H, Moran NA (2005). Evolutionary origins of genomic repertoires in bacteria.. PLoS Biol.

[87] Hacker J, Kaper J (2000). Pathogenicity islands and the evolution of microbes.. Annu Rev Microbiol.

[88] Leach JE, Vera Cruz CM, Bai J, Leung H (2001). Pathogen fitness penalty as a predictor of durability of disease resistance genes.. Annu Rev Phytopathol.

[89] Maddison WP, Maddison DR (2009). Mesquite: a modular system for evolutionary analysis.. http://mesquiteproject.org.

[90] Vinatzer BA, Teitzel GM, Lee MW, Jelenska J, Hotton S (2006). The type III effector repertoire of *Pseudomonas syringae* pv. *syringae* B728a and its role in survival and disease on host and non-host plants.. Mol Microbiol.

[91] Ham JH, Kim MG, Lee SY, Mackey D (2007). Layered basal defenses underlie non-host resistance of Arabidopsis to *Pseudomonas syringae* pv. *phaseolicola*.. Plant J.

[92] Nurnberger T, Lipka V (2005). Non-host resistance in plants: new insights into an old phenomenon.. Mol Plant Pathol.

[93] Lindeberg M, Cartinhour S, Myers CR, Schechter LM, Schneider DJ (2006). Closing the circle on the discovery of genes encoding Hrp regulon members and type III secretion system effectors in the genomes of three model *Pseudomonas syringae* strains.. Mol Plant Microbe Interact.

[94] Yao J, Allen C (2006). Chemotaxis is required for virulence and competitive fitness of the bacterial wilt pathogen *Ralstonia solanacearum*.. J Bacteriol.

[95] Alfano JR, Collmer A (2004). Type III secretion system effector proteins: double agents in bacterial disease and plant defense.. Annu Rev Phytopathol.

[96] Jamir Y, Guo M, Oh H-S, Petnicki-Ocwieja T, Chen S (2004). Identification of *Pseudomonas syringae* type III secreted effectors that suppress programmed cell death in plants and yeast.. Plant J.

[97] Fujikawa T, Ishihara H, Leach JE, Tsuyumu S (2006). Suppression of defense response in plants by the avrBs3/pthA gene family of *Xanthomonas* spp. Mol.. Plant-Microbe Interact.

[98] Fujikawa T, Yamashita T, Tsuyumu S (2006). Hypersensitive response suppression by type III effectors of plant pathogenic bacteria.. J Gen Plant Pathol.

[99] Jackson RW, Athanassopoulos E, Tsiamis G, Mansfield JW, Sesma A (1999). Identification of a pathogenicity island, which contains genes for virulence and avirulence, on a large native plasmid in the bean pathogen *Pseudomonas syringae* pathovar *phaseolicola*.. Proc Natl Acad Sci USA.

[100] Torto-Alalibo T, Collmer CW, Gwinn-Giglio M (2009). The Plant-Associated Microbe Gene Ontology (PAMGO) Consortium: community development of new Gene Ontology terms describing biological processes involved in microbe-host interactions.. BMC Microbiol.

[101] Lindeberg M, Biehl BS, Glasner JD, Perna NT, Collmer A (2009). Gene ontology annotation highlights shared and divergent pathogenic strategies of type III effector proteins deployed by the plant pathogen *Pseudomonas syringae* pv *tomato* DC3000 and animal pathogenic *Escherichia coli* strains.. BMC Microbiol.

[102] Sokurenko EV, Hasty DL, Dykhuizen DE (1999). Pathoadaptive mutations: gene loss and variation in bacterial pathogens.. Trends Microbiol.

[103] Pallen MJ, Wren BW (2007). Bacterial pathogenomics.. Nature.

[104] Arnold DL, Jackson RW, Waterfield NR, Mansfield JW (2007). Evolution of microbial virulence: the benefits of stress.. Trends Genet.

[105] Ma W, Dong F, Stavrinides J, Guttman DS (2006). Type III effector diversification via both pathoadaptation and horizontal transfer in response to a coevolutionary arms race.. PLoS Genetics.

[106] Zhou H, Morgan RL, Guttman DS, Ma W (2009). Allelic variants of the *Pseudomonas syringae* type III effector HopZ1 are differentially recognized by plant resistance systems.. Mol Plant-Microbe Interact.

[107] Yang Y, Gabriel DW (1995). Intragenic recombination of a single plant pathogen gene provides a mechanism for the evolution of new host specificities.. J Bacteriol.

[108] Yang B, Sugio A, White FF (2005). Avoidance of host recognition by alterations in the repetitive and C-terminal regions of AvrXa7, a type III effector of *Xanthomonas oryzae* pv. *oryzae*.. Mol Plant Microbe Interact.

[109] Wichmann G, Ritchie D, Kousik CS, Bergelson J (2005). Reduced genetic variation occurs among genes of the highly clonal plant pathogen *Xanthomonas axonopodis* pv. *vesicatoria*, including the effector gene *avrBs2*.. Appl Environ Microbiol.

[110] Kearney B, Ronald PC, Dahlbeck D, Staskawicz BJ (1988). Molecular basis for evasion of plant host defence in bacterial spot disease of pepper.. Nature.

[111] Gonzalez AI, Ruiz ML, Polanco C (1998). Race-specific insertion of transposable element IS*801* in *Pseudomonas syringae* pv. *phaseolicola*.. Mol Plant Microbe Interact.

[112] Lavie M, Seunes B, Prior P, Boucher C (2004). Distribution and sequence analysis of a family of type III-dependent effectors correlate with the phylogeny of *Ralstonia solanacearum* strains.. Mol Plant Microbe Interact.

[113] Kearney B, Staskawicz B (1990). Characterization of IS*476* and its role in bacterial spot disease of tomato and pepper.. J Bacteriol.

[114] Stavrinides J, Ma W, Guttman DS (2006). Terminal reassortment drives the quantum evolution of type III effectors in bacterial pathogens.. PLoS Pathog.

[115] Mahillon J, Chandler M (1998). Insertion sequences.. Microbiol Mol Biol Rev.

[116] Kim JF, Charkowski AO, Alfano JR, Collmer A, Beer SV (1998). Sequences related to transposable elements and bacteriophages flank avirulence genes of *Pseudomonas syringae*.. Mol Plant Microbe Interact.

[117] Rivas LA, Mansfield J, Tsiamis G, Jackson RW, Murillo J (2005). Changes in race-specific virulence in *Pseudomonas syringae* pv. *phaseolicola* are associated with a chimeric transposable element and rare deletion events in a plasmid-borne pathogenicity island.. Appl Environ Microbiol.

[118] Sundin GW (2007). Genomic insights into the contribution of phytopathogenic bacterial plasmids to the evolutionary history of their hosts.. Annu Rev Phytopathol.

[119] Koebnik R, Kruger A, Thieme F, Urban A, Bonas U (2006). Specific binding of the *Xanthomonas campestris* pv. *vesicatoria* AraC-type transcriptional activator HrpX to plant-inducible promoter boxes.. J Bacteriol.

[120] Ochman H, Moran NA (2001). Genes lost and genes found: evolution of bacterial pathogenesis and symbiosis.. Science.

[121] Pitman AR, Jackson RW, Mansfield JW, Kaitell V, Thwaites R (2005). Exposure to host resistance mechanisms drives evolution of bacterial virulence in plants.. Curr Biol.

[122] Ausubel FM, Brent R, Kingston RE, Moore DD, Seidman JG (1991). Current protocols in molecular biology..

[123] Ciesiolka LD, Hwin T, Gearlds JD, Minsavage GV, Saenz R (1999). Regulation of expression of avirulence gene *avrRxv* and identification of a family of host interaction factors by sequence analysis of *avrBsT*.. Mol Plant Microbe Interact.

[124] Astua-Monge G, Minsavage GV, Stall RE, Davis MJ, Bonas U (2000). Resistance of tomato and pepper to T3 strains of *Xanthomonas campestris* pv. *vesicatoria* is specified by a plant-inducible avirulence gene.. Mol Plant Microbe Interact.

[125] Astua-Monge G, Minsavage GV, Stall RE, Vallejos CE, Davis MJ (2000). Xv4-AvrXv4: a new gene-for-gene interaction identified between *Xanthomonas campestris* pv. *vesicatoria* race T3 and wild tomato relative *Lycopersicon pennellii*.. Mol Plant Microbe Interact.

[126] Staden R, Beal KF, Bonfield JK, Misener S, Krawetz S (2000). The Staden Package.. Methods Mol Biol.

[127] Swofford DL (2003). PAUP*. Phylogenetic Analysis Using Parsimony (*and Other Methods)..

