# Correction: A «Repertoire for Repertoire» Hypothesis: Repertoires of Type Three Effectors are Candidate Determinants of Host Specificity in *Xanthomonas*

**DOI:** 10.1371/annotation/92d243d0-22b2-44da-9618-83b4aa252724

**Published:** 2009-10-09

**Authors:** Ahmed Hajri, Chrystelle Brin, Gilles Hunault, Frédéric Lardeux, Christophe Lemaire, Charles Manceau, Tristan Boureau, Stéphane Poussier

The file for Table S2 is incorrect. Please view the correct file at: 
