# Supplementary material for: Correction: A «Repertoire for Repertoire» Hypothesis: Repertoires of Type Three Effectors are Candidate Determinants of Host Specificity in Xanthomonas
Source: PLoS One. 2009 Oct 9;4(10):10.1371/annotation/92d243d0-22b2-44da-9618-83b4aa252724. doi: 10.1371/annotation/92d243d0-22b2-44da-9618-83b4aa252724 (PMC2763904; doi:10.1371/annotation/92d243d0-22b2-44da-9618-83b4aa252724)
Supplement: Supplementary file 1 [file pone.92d243d0-22b2-44da-9618-83b4aa252724.s001.doc]

Supplemental table S2. List of genes analyzed in this study: gene functions and primers sequences used for PCR amplifications and for making probes for dot-blot hybridizations.

| Gene | Accession number | Confirmed or proposed gene function  (T3E family) | Source strain | Forward primer | Reverse primer | PCR fragment size  (bp) |
| --- | --- | --- | --- | --- | --- | --- |
| *rpoD* | NP_639081 | RNA polymerase sigma-70 factor | XccATCC33913 = CFBP5241 | ATGGCCAACGAACGTCCTGC | AACTTGTAACCGCGACGGTATTCG | 1310 |
| *xopA* | CAJ22071 | unknown function | Xav85-10 = CFBP5618 | CATTGAATACGTCGCACCTCG | GCTGAGGCTAGTGTTGAGCCCTCC | 314 |
| *xopB* | YP_362312 | unknown function | Xav85-10 = CFBP5618 | ATGAAGGCAGAGCTCACACGA | TCAGGCGCGGGTTGGTGCGAAGTA | 1835 |
| *xopC* | CAJ24112 | unknown function | Xav85-10 = CFBP5618 | ATGAAAACAAGTAGTGCAAGC | CTAAAGTTCCGTGATTTTAGATA | 2505 |
| *xopD* | CAJ22068 | SUMO cysteine protease (C48 family) | Xav85-10 = CFBP5618 | ATGGAATATATACCAAGAATGAAGC | CTAGAACTTTTTCCACCACTTGC | 1638 |
| *xopE1* | CAJ21925 | Putative transglutaminase (HopX/AvrPphE family) | Xav85-10 = CFBP5618 | ATGGGACTATGCATTTCAAAGC | TCATCTCGCCACCGTGACAG | 1203 |
| *xopE2* | CAJ23957 | Putative transglutaminase (HopX/AvrPphE family) | Xav85-10 = CFBP5618 | ATGGGGCTATGCAGTTCAAAGC | TCACCAACTCAAGGGTGGGC | 1077 |
| *xopF1* | CAJ22045 | unknown function | Xav85-10 = CFBP5618 | AAACTCTCCAGCGATATCGGCACC | GGCGAAGTGCTTCGCTGTCATGC | 1987 |
| *xopF2* | CAJ24621 | unknown function | Xav85-10 = CFBP5618 | TGAAGCTCCAACGCCAGAACAG | AGCGCCTGCTTCTGACGCAAGG | 1975 |
| *xopJ* | CAJ23833 | cysteine protease (YopJ/AvrRxv family, C55 family) | Xav85-10 = CFBP5618 | GGTCTATGCGTTTCAAAGCCGAGC | ATAGCTGTCTCCAGTGCTGATCGA | 1100 |
| *xopO* | YP_362786 | unknown function | Xav85-10 = CFBP5618 | ATGATCAACACTTCCGTCAAG | TCACCTGTTTATCCGACGAC | 636 |
| *xopN* | CAJ24623 | unknown function | Xav85-10 = CFBP5618 | ATGAAGTCATCCGCATCCGTC | GTGCAGTTCCGTCCCCAAGTTCC | 2157 |
| *xopP* | YP_362967 | unknown function | Xav85-10 = CFBP5618 | TTGAATCGACCAAGCAGGCCCCA | GTGACCGGTCTTTTGGCATGTACG | 1936 |
| *xopQ* | AAV74206 | Putative inosine-uridine nucleoside N-ribohydrolase  (HopQ1-1 family) | Xav85-10 = CFBP5618 | ATGCAGCCCACCGCAATC | TCAGCGCCCGCGTTGCC | 1395 |
| *xopX* | YP_362303 | unknown function  (HopAE1 family) | Xav85-10 = CFBP5618 | ATGGAGATCAAGAAACAGCAAACC | TCAGGACGAAGGCACAGTGC | 2100 |
| *ecf* | AAW88576 | unknown function  (HopAE1 family) | Xav85-10 = CFBP5618 | ATGCAGATCAAAACCGCAGGC | TTATTCCGACTGAGGCACTGG | 2067 |
| *avrRxv* | YP_362202 | cysteine protease (YopJ/AvrRxv family, C55 family) | Xav85-10 = CFBP5618 | ATGTGCGACTCCATAAGAGTGC | TCAGGATTCTAAGGCGTGACG | 1122 |
| *avrRxo1* | CAJ26159 | unknown function | Xav85-10 = CFBP5618 | GTGGGTGTGCGAGTCGC | TCAAATTAGCTCGCTATGAGC | 1353 |
| *avrBsT* | AAD39255 | cysteine protease (YopJ/AvrRxv family, C55 family) | Xav75-3 | TATGCGTTCACTTGGCTTTGG | ATTTTCCTCAATCGAGATGCCTC | 1020 |
| *avrXv3* | AAG18480 | Unknown function | Xav91-118 | ATGACAAGTAGTATCAATCGTTAC | CTACTTAACGAGATTTGTTACG | 657 |
| *avrXv4* | AAG39033 | cysteine protease (YopJ/AvrRxv family, C55 family) | Xav91-118 | ATGAAAAACATATTTAGGTCACTTG | TTAGCTACGACTCAACGCATG | 1080 |
| *avrXacE1* | NP_640642 | Putative transglutaminase (HopX/AvrPphE family) | Xac 306 | ATGGGACTATGCGTTTCAAGGC | TCATCTCGCCACCGTGACAG | 1206 |
| *avrXacE2* | NP_643532 | Putative transglutaminase (HopX/AvrPphE family) | Xac 306 | ATGGGTTGCACTATCTCAACG | TTACTGGCTCTGCTCGCAC | 1071 |
| *avrXacE3* | NP644739 | Putative transglutaminase (HopX/AvrPphE family) | Xac 306 | GTGAGGCGAAGCGAAGCGG | TCACCAACTCAAGGGGGGG | 1143 |
| *XAC3090* | AAM37935 | leucin rich protein (PopC family) | Xac 306 | ATGCCACCGACCGTGGGC | TTACTGATCGCGTGAAGGTGC | 1494 |
| *hpaF* | AAM35284 | leucin rich protein (PopC family) | Xac 306 | ATGTTCAATATAAATCGCTTACTGC | CTAACGGATGTCCCATTCCC | 1941 |
| *pthA1* | NP_644708 | transcription activator-like (AvrBs3/PthA family) | Xac 306 | AGAGCATTGTTGCCCAGTTATCTC | GTTCGGTGACGCCCACTCT | 3381 |
| *avrBs2* | NP_640432 | Putative glycero-P-diester P-diesterase | Xac 306 | GGACTAGTCCTGCCGGTGTTGATGCACGA | CCGCTCGAGCGGTGATCGGTCAACAGGCTTTC | 801 |
| *avrBs1* | NP_637464 | putative tyrosine phosphatase | XccATCC33913 = CFBP5241 | ATGTCCGACATGAAAGTTAATT | TTACGCTTCTCCTGCATTTG | 1338 |
| *avrBs1.1* | AAM41387 | putative tyrosine phosphatase | XccATCC33913 = CFBP5241 | ATGGAGCGGGAGATGGC | TTATGCATTGTGGTCGAGC | 315 |
| *XCC2565* | NP_637913 | leucin rich protein (PopC family) | XccATCC33913 = CFBP5241 | ATGGATAAAAATCTTAATTTGTGG | CTACTGGTGAACCTGGTTC | 1611 |
| *avrXccA1* | AAM43445 | unknown function (AvrXca family) | XccATCC33913 = CFBP5241 | ATGCCCAACGCATCGCCTGCA | CGCAGGTCTGCACCGAACTCAG | 1829 |
| *avrXccA2* | AAM41674 | unknown function (AvrXca family) | XccATCC33913 = CFBP5241 | GTGCTGGAGAGTGCCG | TCATCGGCAGGGTTGTGG | 1488 |
| *avrXccB* | AAM42989 | cysteine protease (YopJ/AvrRxv family, C55 family) | XccATCC33913 = CFBP5241 | GTGGTGGCGGCCCAGAATC | TTAGCTCCAGTACTCGGCGTC | 708 |
| *avrXccC* | NP_637473 | unknown function (AvrB family) | XccATCC33913 = CFBP5241 | ATGTGGTCTCAGCCCG | TTAAATTGGGGGGCGC | 996 |
| *avrXccE1* | NP_636999 | Putative transglutaminase (HopX/AvrPphE family) | XccATCC33913 = CFBP5241 | ATGGGTCTATGCGTTTCG | TCACCAACTCAAGGGCG | 1068 |
